# Shifts in brain dynamics and drivers of consciousness state transitions

**DOI:** 10.3389/fncom.2026.1731868

**Published:** 2026-02-10

**Authors:** Joseph Bodenheimer, Paul Bogdan, Sérgio Pequito, Arian Ashourvan

**Affiliations:** 1 Department of Psychology, The University of Kansas, Lawrence, KS, United States; 2 Ming Hsieh Department of Electrical and Computer Engineering, University of Southern California, Los Angeles, CA, United States; 3 Institute for Systems and Robotics, Instituto Superior Técnico, Universidade de Lisboa, Lisbon, Portugal

**Keywords:** consciousness states, dynamical systems, linear time-invariant model, system identification, unknown inputs

## Abstract

Understanding the neural mechanisms underlying the transitions between different states of consciousness is a fundamental challenge in neuroscience. Thus, we investigate the underlying drivers of changes during the resting-state dynamics of the human brain, as captured by functional magnetic resonance imaging (fMRI) across varying levels of consciousness (awake, light sedation, deep sedation, and recovery). We deploy a model-based approach relying on linear time-invariant (LTI) dynamical systems under unknown inputs (UI). Our findings reveal distinct changes in the spectral profile of brain dynamics—particularly regarding the stability and frequency of the system's oscillatory modes during transitions between consciousness states. These models further enable us to identify external drivers influencing large-scale brain activity during naturalistic auditory stimulation. Our findings suggest that these identified inputs delineate how stimulus-induced co-activity propagation differs across consciousness states. Notably, our approach showcases the effectiveness of LTI models under UI in capturing large-scale brain dynamic changes and drivers in complex paradigms, such as naturalistic stimulation, which are not conducive to conventional general linear model analysis. Importantly, our findings shed light on how brain-wide dynamics and drivers evolve as the brain transitions toward conscious states, holding promise for developing more accurate biomarkers of consciousness recovery in disorders of consciousness.

## 1 Introduction

Understanding the intricate neural mechanisms governing transitions between various states of consciousness is a key challenge within neuroscience. The complexity of the brain, with its myriad interactions and dynamic states, highlights the difficulty in unraveling these processes. With the advent of modern neuroimaging techniques, particularly functional magnetic resonance imaging (fMRI), unprecedented access to whole-brain activity and its transitions between different consciousness states has emerged (Boly et al., 2007; Coleman et al., 2009; Lloyd, 2002; Soddu et al., 2009). However, the neural underpinnings of large-scale brain dynamics and the interplay between cortical and subcortical regions across different consciousness states remain elusive.

Early investigations employing univariate analyses of functional and metabolic brain activity have revealed extensive changes in brain function across different consciousness states (Nakayama et al., 2006). Pioneering neuroimaging studies have demonstrated that anesthetics such as propofol produce dose-dependent, bilateral reductions in activity within the thalamus, midbrain reticular formation, cuneus-precuneus, posterior cingulate cortex, prefrontal cortices, and parietal associative cortices (Fiset et al., 1999; Kaisti et al., 2003). However, this univariate approach provides limited insights due to the brain's complex network interactions underlying consciousness (Bagshaw and Khalsa, 2013; Crone and Monti, 2018).

Early evidence for network-level dysfunction during anesthesia emerged from two key observations. First, task-based studies revealed impaired processing in various domains, including visual (Heinke and Schwarzbauer, 2001), auditory (Heinke et al., 2004; Kerssens et al., 2005; Plourde et al., 2006), verbal (Fu et al., 2005), emotional (Paulus et al., 2005), and memory (Sperling et al., 2002; Honey et al., 2005). Second, higher-order association areas, responsible for complex processing, were found to be more sensitive to the effects of anesthesia compared to lower-order regions involved in basic processing (Dueck et al., 2005; Heinke and Koelsch, 2005; Ramani et al., 2007).

Network neuroscience effectively investigates brain dynamics by examining changes in functional and structural connectivity, with the resting-state paradigm providing key insights into baseline functional activity across various consciousness states (Sporns, 2011; Luppi et al., 2021; Crone et al., 2020; Demertzi et al., 2019). Patients in unresponsive wakefulness syndrome and minimally conscious state show decreased functional connectivity (FC) in regions related to default mode network (DMN) as well as executive control network (ECN) and auditory network when compared to healthy controls (Demertzi et al., 2014). Patients showed a decrease in FC distributed in the parietal cingulate cortex, precuneus, lateral parietal cortex, and medial prefrontal cortex (Wu et al., 2015). Studies have shown that restoration of thalamo-frontal connectivity can also serve as predictive markers for transitions toward conscious states (Crone et al., 2018). Studies using anesthesia-induced transitions also show specific brain circuits like those involving the thalamus and large-scale networks like the DMN are crucial for wakefulness. Anesthetics progressively disrupt connectivity within these networks (i.e., DMN, ECN) at higher doses, while lower-order sensory networks remain somewhat functionally connected (Deshpande et al., 2010; Stamatakis et al., 2010; Greicius et al., 2008; Boveroux et al., 2010). This suggests that while basic sensory processing might persist, integrating information across brain regions is impaired under anesthesia, potentially due to disrupted subcortical thalamic regulation (Mhuircheartaigh et al., 2010).

While the resting-state paradigm offers insights into system-wide changes, understanding the brain's different states requires examining responses to external stimuli. Studies using transcranial magnetic stimulation show that perturbation spread varies with the conscious state, highlighting the role of thalamocortical circuitry (Sarasso et al., 2014). More complex stimuli can further reveal network engagement; for example, auditory processing areas show varying activation patterns in response to musical stimuli under different levels of propofol-induced sedation (Dueck et al., 2005), and FC is disrupted during auditory word listening under propofol-induced sedation (Liu et al., 2012).

Machine learning (ML) has become a powerful tool for studying consciousness, with techniques like artificial neural networks revealing activation patterns in networks associated with wakefulness and arousal (Khosla et al., 2019; Perl et al., 2020). However, while these approaches excel at prediction, they often lack mechanistic explanations for how brain networks transition between states (Jin et al., 2023). To bridge this gap, computational modeling approaches aim to reveal the mathematical underpinnings of neuronal activity (Luppi et al., 2023). This allows researchers to explore how FC dynamically changes across consciousness states. Generative models focus on capturing the statistical properties of the data, while dynamical models examine how changes in these systems lead to transitions in consciousness (Luppi et al., 2022a, 2023, 2022b; Kandeepan et al., 2020). By combining these techniques, we can gain a deeper understanding of the intricate network dynamics that govern consciousness.

In this work, we capitalize on a publicly available dataset from Kandeepan et al. (2020), which measures resting-state dynamics in response to naturalistic auditory stimulation across different consciousness states—wakefulness, light sedation, deep sedation, and recovery. We employ our recently developed computational framework to identify the large-scale oscillatory modes of the brain and the unknown external drivers influencing these dynamics (Ashourvan et al., 2022). Our results demonstrate the stabilization of several oscillatory modes overlapping transmodal cortices during resting-state scans. The examination of auditory stimulation scans also reveals that these unknown inputs uncover task-specific, spatiotemporally overlapping patterns of consciousness-dependent co-activation and deactivation, which drive brain-wide dynamics. Our findings underscore the utility of this approach in characterizing brain dynamics and their responses to stimuli, providing novel insights into consciousness dynamics and potential applications in forecasting consciousness recovery, particularly in disorders of consciousness patients.

## 2 Materials and methods

### 2.1 Dataset and pre-processing

We used a publicly available dataset from Kandeepan et al. (2020), which was accessed from Openneuro.org (Kandeepan et al., 2020). The dataset protocol included 17 healthy participants (4 women; mean age = 24 ±5) with no history of neurological disorders. Participants completed fMRI at four levels of sedation (awake, mild sedation, deep sedation, and recovery) during resting-state scans as well as while listening to a 5-min audio recording from the movie “Taken.” Functional echo-planar images (EPI) were acquired at a matrix size of 64 × 64 with a spatial resolution of 3 mm isotropic voxels. Images contain 33 slices with a 25% inter-slice gap with a repetition time (TR) of 2,000 ms and time echo (TE) of 30 ms. Audio task and resting-state scans had 155 and 256 samples, respectively. An anatomical scan was also obtained using a T1-weighted 3D MPRAGE (magnetization prepared rapid gradient echo) sequence. Anatomical image acquired at a matrix size of 240 × 256 × 192 with a spatial resolution of 1 mm isotropic voxels and 4,250 ms TE.

The dataset obtained from Openneuro was preprocessed through the fMRIprep preprocessing pipeline. T1w images in the data were used to create a reference T1w to correct for intensity non-uniformity with N4BiasFieldCorrection (ANTs) (Tustison et al., 2010). The reference was then skull-stripped using a NiPype implementation of the antsBrainExtraction.sh (ANTs) workflow tool using the OASIS brain extraction template (Marcus et al., 2007) as a target. Brain surfaces were reconstructed from the reference T1w image using the FreeSurfer tool recon-all (Dale et al., 1999). Brain tissue segmentation of gray matter, white matter, and cerebrospinal fluid was computed from the reference T1w image using FSL's FAST (Zhang et al., 2001), spatial normalization to the ICBM Nonlinear Asymmetrical template (MNI152NLin2009cAsym) was performed using antsRegistration (ANTs).

For BOLD images (EPI), a reference image was created from the median of motion-corrected BOLD images. Head motion is estimated using FSL's mcflirt (Jenkinson et al., 2002). The BOLD runs were then slice-timing corrected using AFNI's 3dTshift (Cox and Hyde, 1997) and underwent susceptibility distortion correction (SDC). These files are then aligned using the gray/white-matter boundary and resampled to MNI152NLin2009cAsym and fsaverage (Zhang et al., 2001) (Freesurfer) template space.

The preprocessed BOLD images underwent further processing using the eXtensible Connectivity Pipeline-DCAN (XCP-D) postprocessing pipeline (Mehta et al., 2023). Postprocessing denoising of the data included confound regression of nuisance regressors using the 36P strategy configuration, which includes six realignment motion parameters, white matter, CSF, and global signal parameters. To retain as much data in the final output, temporal censoring and data filtering were disabled. For the output final step, minimum coverage was set to 0.01. We excluded 2 participants with any ROIs that did not meet this criteria. Voxel-wise time series were extracted from the denoised BOLD images and parcellated to the combined 4S atlas (Cieslak et al., 2021). From this combined atlas, we utilized the Schaefer cortical atlas (Schaefer et al., 2017) at 100-region resolution for analysis.

### 2.2 Linear time-invariant (LTI) dynamical systems with external inputs

Each region of interest *i* provides a time series denoted by *x*_*i*_[*k*] at sampling point *k* = 0, …, *T*. We consider a total of *n* = 100 cortical ROIs. These signals are collectively represented by the vector $x\left[k\right]={\left[x_{1}\left[k\right],\;\ldots x_{n}\left[k\right]\right]}^{⊺}$, with *k* = 0, …, *T*, referred to as the state of the system, describing the BOLD signal's evolution across regions. The system's state evolves primarily due to (*i*) cross-dependencies among signals from different regions and (*ii*) external inputs, which may be excitation noise or unaccounted extrinsic stimuli.

To model the system's state evolution, we propose


$$\begin{array}{l} x\left[k+1\right]=Ax\left[k\right]+Bu\left[k\right]+\omega_{k},\;k=0,\ldots ,T, \end{array}$$ (1)

where *A*∈ℝ^*n*×*n*^ represents the coupling dynamics, *B*∈ℝ^*n*×*p*^ is the input matrix describing the influence of inputs *u*[*k*]∈ℝ^*p*×1^ on state evolution, and $\omega_{k}\in {\mathbb{R}}^{n}$ is internal dynamics noise at sampling point *k*. Notably, ${\left\{x\left[k\right]\right\}}_{k=0}^{T}$ denotes BOLD signals across ROIs, being the only known information. However, the underlying neural activity state remains unknown due to the absence of the hemodynamic response function in our model. Hence, the input in the model reflects external drivers of regional BOLD, indirectly capturing neural activity. To determine the system parameters (Equation 1) (*A*, *B*, ${\left\{u\left[k\right]\right\}}_{k=0}^{T}$), we minimize the distance between the system's state *x*[*k*] and the estimated state $\hat{x}\left[k\right]$ driven by unknowns, yielding the optimization problem:


$$\begin{array}{l} {\left\{\hat{x}\left[k\right]\right\}}_{k=0}^{T}\in \arg\underset{z\left[0\right],\ldots ,z\left[T\right]}{\min}\;\|z\left[k\right]-x\left[k\right]{\|}_{2}^{2}\\ \text{ s.t. }z\left[k+1\right]=Az\left[k\right]+Bu\left[k\right]. \end{array}$$ (2)

This problem is more complex than standard least squares due to unknown system parameters (Ljung, 1999). Therefore, following Gupta et al. (2018), we undertake the following steps: (i) setting *z*[0] = *x*[0] and ${\left\{u\left[k\right]\right\}}_{k=0}^{T}$ to zero to approximate *A*; (ii) assuming *A* from step (i), providing a sparse low-rank structure to *B* to approximate *z*[0] and ${\left\{u\left[k\right]\right\}}_{k=0}^{T}$, yielding subsequent *z*[0], …, *z*[*T*], ${\left\{\hat{x}\left[k\right]\right\}}_{k=0}^{T}$; and (iii) assuming ${\left\{z\left[k\right]\right\}}_{k=0}^{T}$ and ${\left\{u\left[k\right]\right\}}_{k=0}^{T}$ as approximated, obtaining an approximation for *B*. Steps (ii) and (iii) are performed iteratively until the parameter estimates converge (typically within a few iterations). To prevent the external inputs from solely capturing all the information, we penalize their use in the optimization objective function. This is achieved by adding a regularization factor (i.e., sparsity term, $||z\left[k\right]-x\left[k\right]|{|}_{2}^{2}+\lambda ||u|{|}_{1}+\lambda ||B|{|}_{1}^{2}$ with weight λ>0) that discourages overly complex input patterns. For detailed algorithmic procedures, refer to SI1.

We demonstrated in our previous work that unaccounted external inputs result in errors in the estimation of system matrix *A* (Ashourvan et al., 2022). Therefore, in a modified version of this algorithm, in step (i), we estimate *A* from *x*[*k*] measured during resting-state scans (i.e., an extended period without task-related external stimulation). Next, we iteratively repeat steps (ii) and (iii) as detailed above.

The shorter resting-state scans in the Kandeepan et al. (2020) dataset posed limitations on individual-level parameter estimation. To overcome this constraint, rather than computing separate system matrices based on the LTI model for each subject, we derived a single group-level system parameter for each consciousness level. This was achieved through simultaneous minimization of the least squared error across all participants using unconstrained nonlinear optimization employing a quasi-Newton algorithm (Broyden, 1970; Shanno, 1970).

For the mathematical description of the cost function for the explained optimization problem of estimating a single *A* matrix of an autonomous LTI system without external inputs using the least squared error across all subjects simultaneously, we can represent it as follows: Given a set of observations *x*_*i*_ for *N* subjects over *T* time points and a model prediction ${\hat{x}}_{i}$ based on the LTI system with a system matrix *A*, the cost function can be defined as the sum of squared errors across all subjects:


$$\begin{array}{l} \;J\left(A\right)=\overset{N}{\underset{i=1}{\sum }}\overset{T}{\underset{k=1}{\sum }}{\left(x_{i}\left[k\right]-{\hat{x}}_{i}\left[k\right]\right)}^{2}\\ \text{s.t.}\;{\hat{x}}_{i}\left[k+1\right]=A{\hat{x}}_{i}\left[k\right]+Bu\left[k\right], \end{array}$$ (3)

where *x*_*i*_[*k*] represents the observed data for subject *i* at time *k*, and ${\hat{x}}_{i}\left[k\right]$, is the model prediction based on the LTI system with system matrix *A*.

The optimization problem is then to find the system matrix *A* that minimizes this cost function: $\underset{A}{\min}J\left(A\right)$. This optimization is performed using iterative algorithms such as the quasi-Newton method mentioned earlier, which iteratively updates the estimate of *A* until convergence to a minimum of the cost function is achieved.

Since we did not know the true dimensionality of the external inputs, we approximated the dimensions of the input matrix *B* by performing principal component analysis on the residuals of the models. As seen in Supplementary Figure 4, the first 10 and 25 PCs capture more than ≈70% and ≈90% of variance in the average residuals across all tasks, respectively.

In addition, we demonstrate that we identify external input patterns during the auditory stimulation task and consciousness levels similarly at both low and high-dimensional input matrices (Supplementary Figure 7). Therefore, we select *p* = 10 for input matrix *B* to estimate the inputs in the main manuscript to capture the large-scale cortical input patterns.

### 2.3 Eigenmode decomposition for brain dynamics analysis

Our analysis leverages the concept of eigenmode decomposition to understand the dynamic behavior of the brain's BOLD signal. Given an LTI description of the system dynamics, we can decompose the evolution of this system into its eigenmodes.

#### 2.3.1 Eigenmodes and their properties

An eigenmode is characterized by an eigenvalue-eigenvector pair (λ_*i*_, *v*_*i*_). The system dynamics satisfy the equation *Av*_*i*_ = λ_*i*_*v*_*i*_, where *A* is the system matrix, *v*_*i*_ is the eigenvector corresponding to the eigenvalue λ_*i*_. Each eigenmode describes the oscillatory behavior of the system along a specific direction defined by the eigenvector *v*_*i*_.

The eigenvalue λ_*i*_ itself holds valuable information about the dynamics in that direction:

- **Frequency**: Represented in polar coordinates by (θ_*i*_, |λ_*i*_|), the frequency of the oscillation associated with the eigenmode is
$$\begin{array}{l} f_{i}=\frac{\theta_{i}}{2\pi }\delta t, \end{array}$$ (4)
  where δ*t* is the sampling frequency of the data.
- **Stability** (Damping Rate): The Stability or time scale, which reflects how quickly the oscillation decays or grows over time, is captured by
$$\begin{array}{l} \rho_{i}=\frac{\log\left(\left|\lambda_{i}\right|\right)}{\delta t}. \end{array}$$ (5)

The interpretation of the time scale depends on the magnitude of the eigenvalue:

- *Damping (Stable)*: If |λ_*i*_| < 1, the magnitude of the oscillation along that direction decays to zero over time, indicating a stable process;
- *Growing (Unstable)*: If |λ_*i*_| > 1, the magnitude of the oscillation grows without bound, indicating an unstable process; and
- *Meta-Stable*: If |λ_*i*_|≈1, the process oscillates between periods of stability and instability, exhibiting a meta-stable behavior.

#### 2.3.2 From eigenvectors to spatial contributions

The eigenvector matrix, denoted by *V* = [*v*_1_, …, *v*_*n*_], contains all the eigenvectors as columns. We can express the system dynamics in terms of these eigenvectors using a change of variable:


$$\begin{array}{l} z\left[k\right]=V^{*}x\left[k\right] \end{array}$$ (6)

Here, V^*^ is the conjugate transpose of *V*, *x*[*k*] is the original state vector of the system at time step *k*, and *z*[*k*] is the transformed state vector. The *i*^*th*^ component of *z*[*k*], denoted by *z*_*i*_[*k*], represents a weighted combination of the original state variables based on the *i*^*th*^ eigenvector, *v*_*i*_. Therefore, *z*_*i*_[*k*] captures the specific spatial contributions of the different ROIs to the overall brain activity at the spatiotemporal frequency characterized by the eigenvalue λ_*i*_.

The variable *z*[*k*] is introduced as a coordinate transformation into the eigenmode basis of the system matrix for modal interpretation, with all subsequent analyses performed implicitly in this space and without imposing any additional dimensionality reduction. By analyzing the eigenmodes of the system dynamics, we can extract key information about the brain's BOLD signal evolution. The eigenvalues reveal the timescales of the underlying processes, while the eigenvectors describe the spatial contributions of different ROIs. Together, this decomposition provides a comprehensive understanding of the spatiotemporal dynamics of brain activity.

### 2.4 Statistics

#### 2.4.1 Identifying shared eigenmode profiles

We employed k-means clustering to identify groups of eigenmodes exhibiting similar spatial profiles across all consciousness states. Calinski-Harabasz (Caliński and Harabasz, 1974), Davies-Bouldin (Davies and Bouldin, 1979), and Silhouette (Rousseeuw, 1987) criteria were used to assess the optimal clustering resolution (see SI2 for details). However, these criteria yielded inconsistent results, suggesting that the eigenvector clusters lack a well-defined optimal number of communities at a specific topological scale. The elbow method, which analyzes explained variance vs. the number of clusters, further confirmed the absence of a clear optimal clustering resolution (Supplementary Figure 1). Therefore, we adopted a data-driven approach by systematically exploring cluster solutions ranging from k = 3 to k = 20. For each k, we repeated the clustering procedure 100,000 times to identify clusters that reliably detected consciousness state-dependent effects.

To ensure consistent eigenvector ordering across clustering solutions, we employed the Hungarian algorithm (Munkres, 1957) to optimally match cluster centroids. For each clustering iteration, we computed a pairwise similarity matrix between the current cluster centroids and a reference set of centroids using Pearson correlation coefficients. Since the Hungarian algorithm minimizes cost, we negated the correlation values to create a cost matrix where higher similarity corresponded to lower cost. The algorithm then determined the optimal one-to-one assignment that maximized the total similarity between matched centroid pairs. This assignment was used to consistently reorder cluster centroids, reassign cluster labels, and reorder all associated k-means outputs to match the reference ordering. This approach resolved the label-switching problem inherent in clustering algorithms and enabled meaningful comparison of eigenvector assignments across iterations and analysis conditions.

Subsequently, we examined potential differences in eigenvalue stability and frequency across these clusters. For each cluster and iteration, we tested whether the distribution of eigenvalues differed significantly across the four consciousness states using Analysis of Variance (ANOVA) with a significance threshold of *p* < 0.05. We reported effect sizes using Cohen's f and applied false discovery rate (FDR) correction for multiple comparisons across all iterations and clusters. To identify robust state-dependent effects, we calculated the percentage of iterations showing significant results after FDR correction as a proxy for reliability.

To quantify the reliability and consistency of the identified cluster structures, we performed two complementary analyses across the 100,000 iterations. First, we computed the percentage of variance explained by the first principal component of all centroids within each cluster, where higher values indicate consistent spatial patterns with minimal variation. Second, we calculated Pearson correlations between each cluster centroid and its corresponding reference centroid from the first iteration (used as the Hungarian sorting template), where higher correlations reflect successful preservation of cluster identity across iterations. Together, these metrics quantified both within-cluster consistency and cross-iteration stability of the identified spatial patterns.

To identify estimated inputs reflecting changes corresponding to different consciousness levels, we conducted a principal component analysis (PCA) on the concatenated spatial profiles (*B*) of all estimated inputs across all subjects for each consciousness state. Subsequently, we determined a single input with the highest absolute principal component (PC) loading for each component. Inputs with negative PC loadings were multiplied by −1. Next, for each ROI, we performed an ANOVA to assess the significance of differences across the means among the four consciousness levels (*p* < 0.05). We corrected the calculated test statistics for multiple comparisons across all ROIs using the FDR method (Benjamini and Hochberg, 1995).

#### 2.4.2 Consciousness state classification

We implemented a Linear Support Vector Machine (SVM) classifier to predict the consciousness state based on the vectors from the spatial input matrix *B* associated with the first four leading PCs concatenated *B* matrices across all subjects. The training process involved several preprocessing steps to ensure the data was suitable for modeling. Initially, we applied PCA to feature sets to the numeric predictor variables to reduce the dimensionality of the data. We retained enough principal components to explain 95% of the variance in the predictor data. This reduction helped enhance computational efficiency and improve the model's performance by focusing on the most significant features.

We trained the SVM classifier with the preprocessed data, which involved defining a prediction function to enable future predictions on new data. The performance of the trained classifier was evaluated using five-fold cross-validation, yielding a validation accuracy that reflects the model's predictive capability. The trained classifier and its validation accuracy were then outputted for further use and assessment.

The ROC curves for each class label were generated by computing the true positive rate and false positive rate for various threshold settings. By plotting these rates, we create ROC curves that visually represent the model's performance in distinguishing between classes. The area under each ROC curve (AUC) indicates the model's ability to correctly classify instances of each class, with higher AUC values signifying better performance.

## 3 Results

This section explores the intricate relationship between brain activity and consciousness levels, examining how shifts from wakefulness to deep sedation manifest in the brain's dynamic oscillations and responses to external stimuli. In Section 3.1, a linear time-invariant model under unknown inputs is employed to dissect the spectral fingerprints of brain activity across various consciousness states. Building upon this, Section 3.2 investigates how the brain's response to auditory stimuli changes across these states. The identified patterns of brain activity serve as a basis for classifying consciousness levels, demonstrating the potential for novel diagnostic and monitoring tools in this field.

### 3.1 LTI systems' spectral fingerprints of consciousness

To capture and describe large-scale oscillatory patterns in brain activity, we applied LTI system identification to estimate group-level system dynamics parameters for each consciousness level (see Materials and Methods). Eigendecomposition of the estimated system parameters revealed the spatiotemporal patterns of oscillatory modes within the modeled resting-state brain dynamics.

To investigate how consciousness level transitions (from awake to deep sedated states) alter the system's spectral profile, we performed k-means clustering on the spatial components (eigenvectors) of eigenmodes across all consciousness states simultaneously. Clustering served as a population-level summarization tool to group eigenmodes with similar spectral and stability properties across subjects, rather than to define discrete mechanistic mode classes. Detailed analysis using several clustering quality metrics (Supplementary Figure 1) revealed no single optimal cluster size at which eigenvectors naturally partition. Therefore, we systematically evaluated k-means clustering solutions with k ranging from 3 to 20 to identify the most stable patterns.

**Figure 1:**
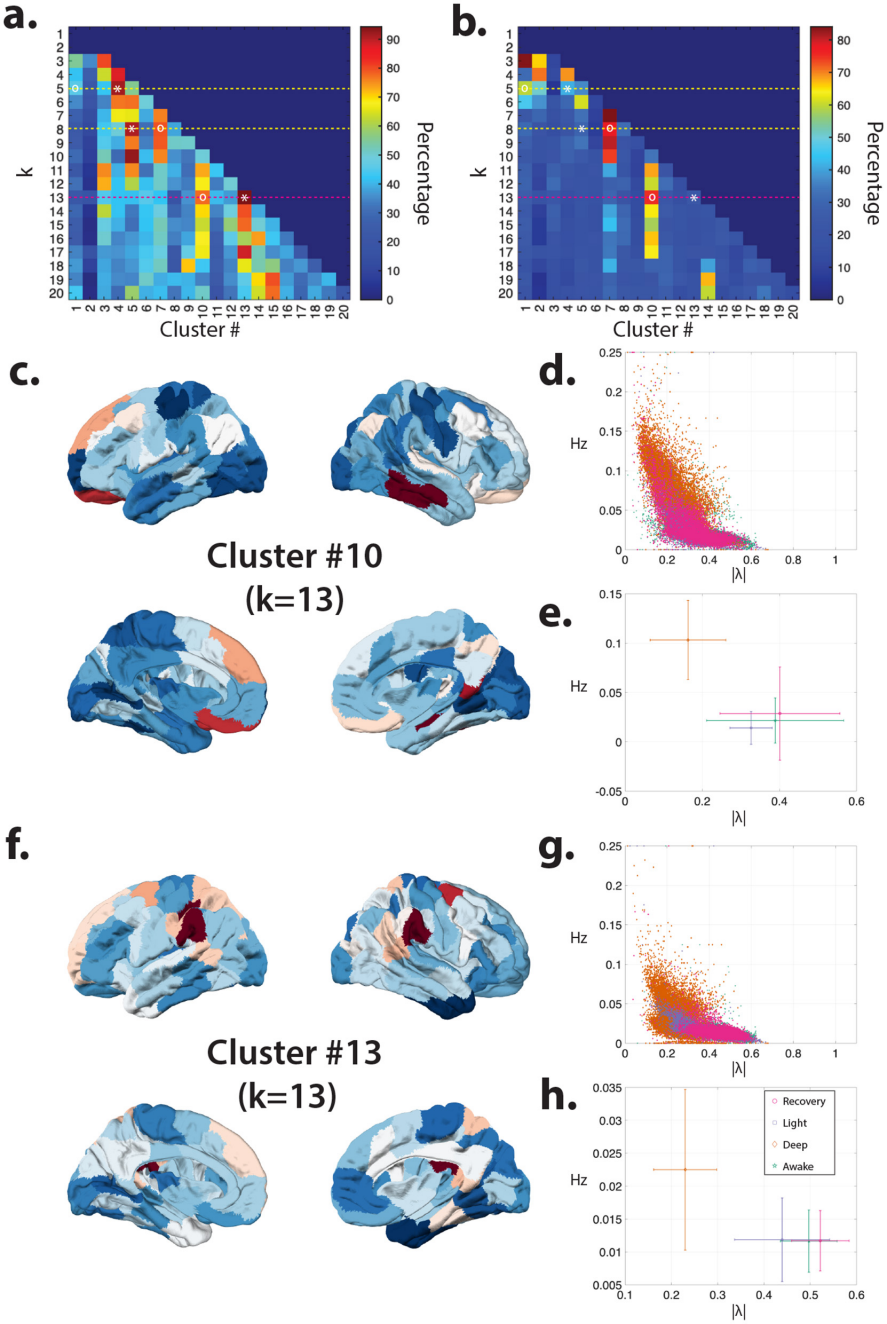
Identifying eigenmode clusters with consciousness-state–dependent effects. **(a)** Percentage of iterations (out of 100,000 repetitions) in which ANOVA revealed significant differences (*p* < 0.05, FDR-corrected for multiple comparisons) in eigenmode stability (|λ|) across consciousness states for each cluster. Cluster centroids are sorted from smallest to largest k using the Hungarian algorithm (see Methods). **(b)** Same as **(a)**, but for eigenmode frequency (angle) instead of stability. Dashed lines indicate cluster resolutions (k = 5, 8, and 13) that produce peak discriminability in both stability and frequency across consciousness states. Two clusters with the largest differences in frequency and stability are marked with “*” and “o.” Similar cluster centroids are consistently identified across different k values; Supplementary Figure 3 shows their similarity across clustering resolutions. **(c, f)** spatial centroids of the two clusters are marked in **(a, b)** for the k = 13 solution. The brain overlay highlights regions with greater contributions to the eigenvector cluster centroid using warm (red) colors. **(d, g)** Distribution of mean eigenvalues' frequency and stability for the clustered eigenmodes across all 100,000 iterations, shown separately for each cluster. **(e, h)** Mean and standard deviation of eigenvalues across iterations for each consciousness state within the corresponding clusters.

To assess cluster reliability and identify robust changes across consciousness states, we repeated k-means clustering 100,000 times for each k value and tested the significance of changes in the distribution of all the eigenvalues' stability and frequency within each cluster using ANOVA. Figures 1a, b demonstrates that as cluster number increases, certain clusters exhibit robustly significant changes in both stability and frequency across consciousness states, with these effects remaining consistent across more than 60-80% of repetitions. Specifically, Figures 1c–h displays the mean centroids of the most prominent clusters showing robust reductions in mode stability, particularly during deep sedation. We further validated cluster stability across repetitions by calculating (1) the percentage of variance explained by the first principal component across all 100,000 Hungarian algorithm-sorted (Munkres, 1957) k-means solutions and (2) the correlation between sorted cluster solutions (see Supplementary Figure 4 for details).

**Figure 2:**
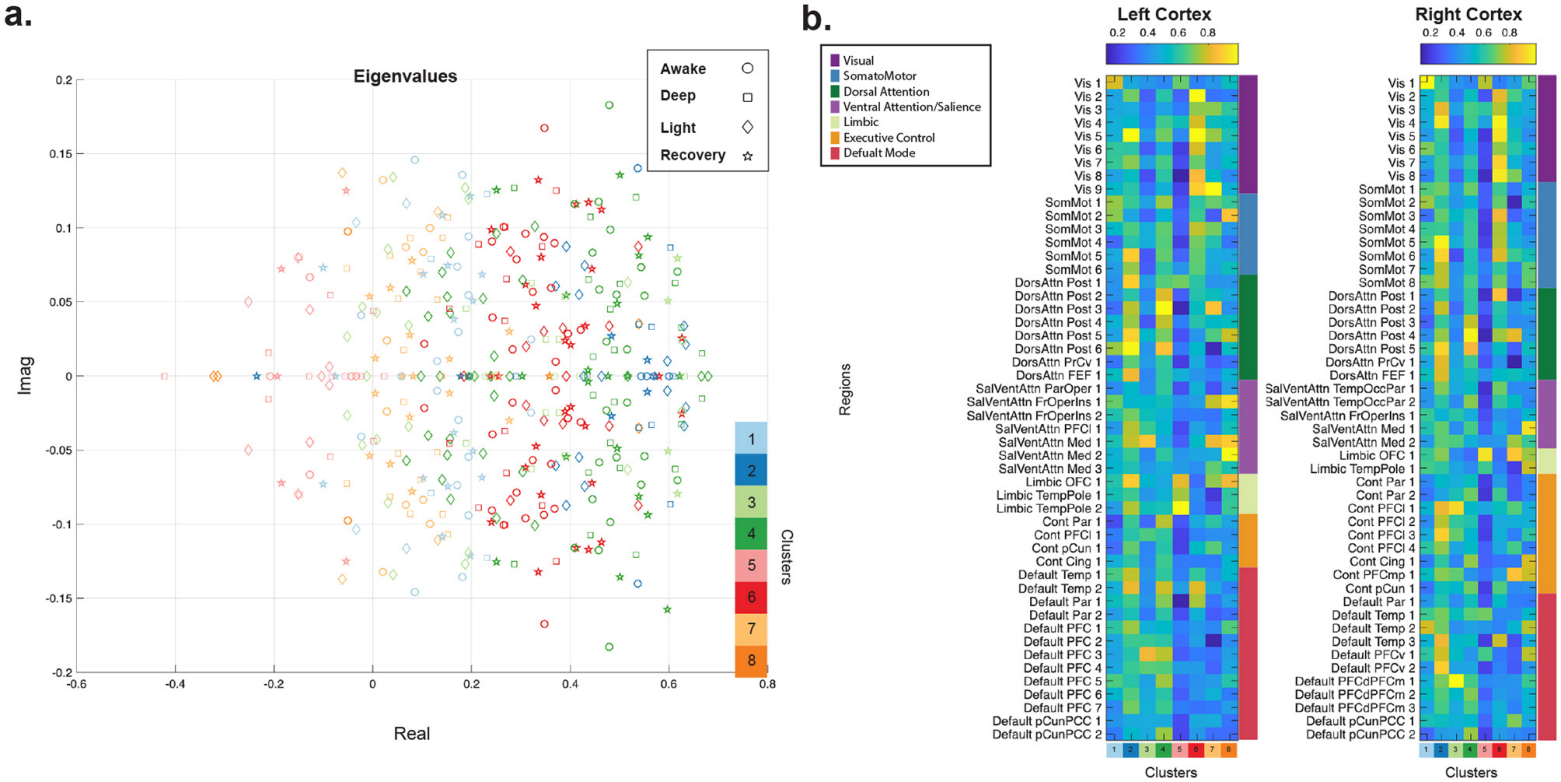
Spectral profiles of the brain's oscillatory modes at different levels of consciousness. **(a)** Distribution of eigenvalues from resting-state scans across varying states of consciousness. The identified eigenvector clusters (*k* = 8) are represented with distinct colors, and different consciousness states (awake, light sedation, deep sedation, and recovery) are denoted by different markers. **(b)** Identified centroid of eigenvector clusters (color-coded column-wise) associated with the eigenvalue from panel **(a)**.

**Figure 3:**
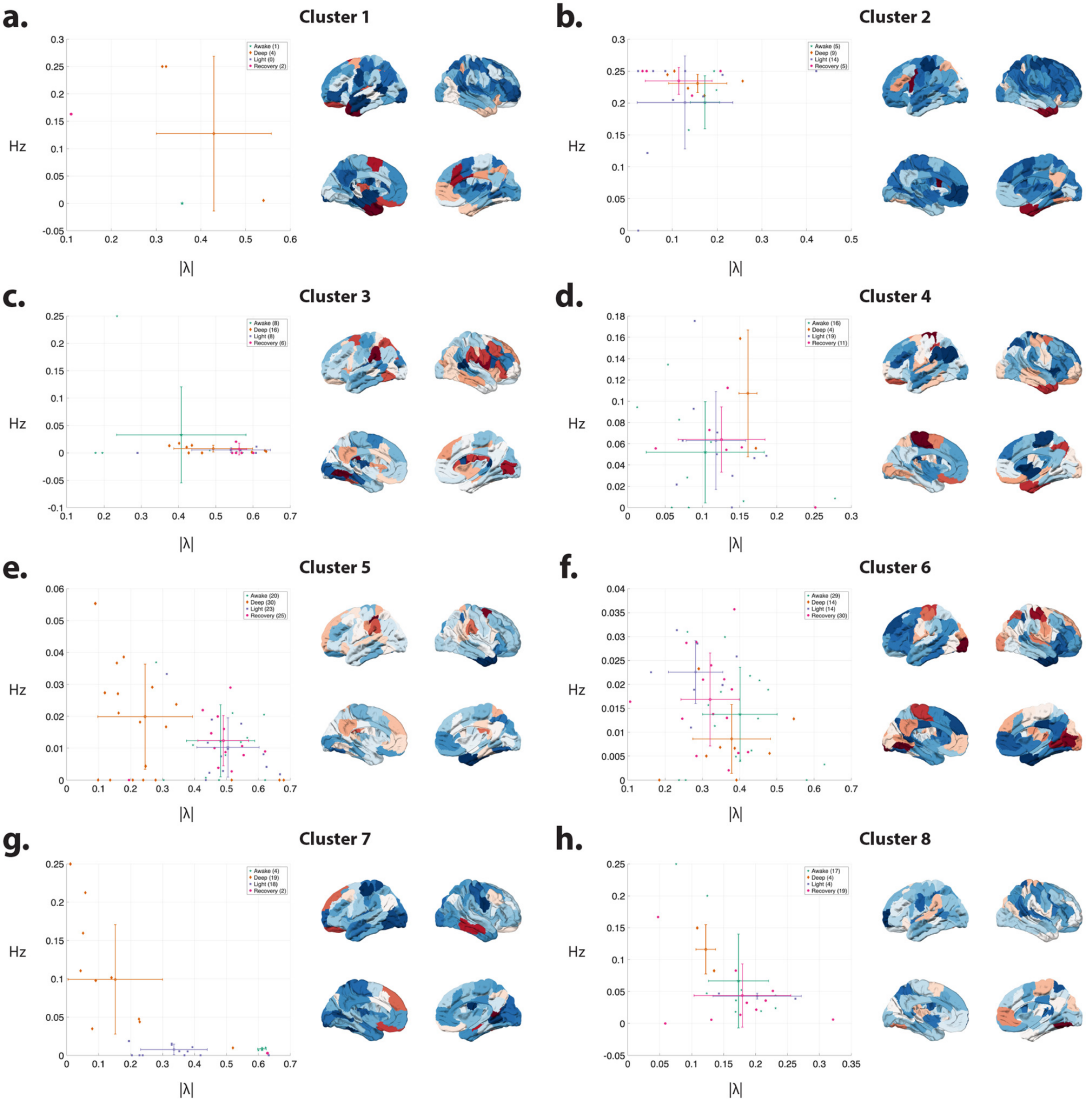
Sedation-induced changes in the stability and frequency of eigenmodes. **(a–f)** Frequency (Hz) and stability of eigenvalues derived from group-level system dynamics for each consciousness level, grouped by *k*-means clusters (k = 8) identified in Figure 2. The bar plots represent the mean and standard deviation of frequency and stability values for each state of consciousness. The inset legend indicates the number of eigenvalues associated with each consciousness state from the total number of all eigenvalues across 4 consciousness level states (i.e., 4 states × 100 eigenvalues = 400). ANOVA results examining differences in frequency and stability across the four states for each cluster are reported in Supplementary Figure 3. The warm (red) colors on brain overlays highlight regions with greater contributions to the eigenvector cluster centroids.

We identified several cluster resolutions that yielded the highest discrimination between consciousness states, including k = 5, 8, and 13 (Figure 1a). Supplementary Figure 3 demonstrates that these key clusters remain consistently identifiable across increasing clustering resolutions. Figure 2 presents the eigenvalue distributions and spatial profiles of identified cluster centroids for a representative k = 8 solution from a single k-means run. Notably, although clustering was performed on eigenvectors (spatial patterns), the eigenvalues associated with each cluster were also organized systematically by spectral profile. Figure 3 illustrates the distribution of frequency and stability values for eigenvalues within each cluster for this representative solution. Closer examination of the identified clusters revealed distinct changes in the number, frequency, and stability of eigenmodes across consciousness levels. The most pronounced effect was a systematic reduction in mode stability during deep sedation, particularly within clusters 5 and 7. These findings demonstrate the effectiveness of this approach in capturing identifiable spectral signatures associated with altered states of consciousness.

### 3.2 Consciousness state-dependent co-activation

Leveraging our framework with unknown inputs, we investigate how brain responses to auditory stimuli vary across consciousness states. Specifically, it enables the extraction of both spatial (*B*) and temporal (*U*) profiles of external inputs influencing brain activity – see Materials and Methods for details. In fact, we hypothesize that the identified input patterns will capture the spread and influence of the stimuli on brain co-activation across consciousness levels.

First, we considered a lower dimensionality for the inputs (*p* = 10) and applied a moderate level of regularization (λ = 0.5) to effectively capture the large-scale driver patterns. This decision stemmed from our analysis, where examining the residuals of the LTI model without external input via principal component analysis (PCA) revealed that approximately 70% of the variance of the residuals could be explained by just ten components (Supplementary Figure 4).

**Figure 4:**
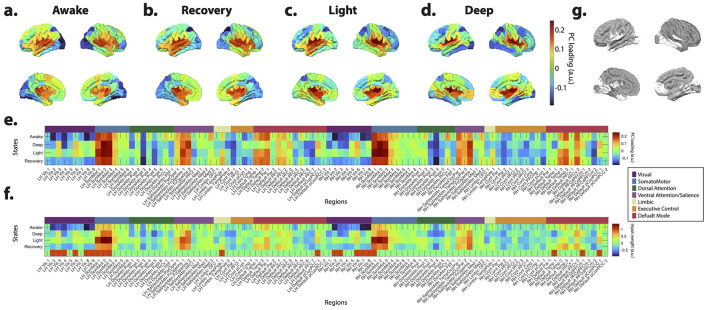
Principal component analysis (PCA) of the inputs' spatial profiles estimated during auditory stimulation paradigm. **(a–d)** The third principal component (PC3) of the inputs' spatial profiles, estimated during auditory stimulation, shows consistent patterns across different states of consciousness. PC3 captures the activation of the auditory cortex and other active regions. **(e)** The Scheafer 100 ROI brain atlas (Schaefer et al., 2017) labels, as well as the seven resting state brain networks identified by Yeo et al. (2011) corresponding to the PC3 in **(a–d)**. **(f)** Group-average spatial profiles of the input matrix (*B*) corresponding to the component with the highest PC3 loading for each subject. For each subject, the *B* pattern associated with the maximal PC3 loading was identified and then averaged across subjects within each state. While the resulting spatial profiles are broadly similar across states, regional differences are evident. ANOVA identifies brain regions exhibiting significant state-dependent effects (*p* < 0.05, FDR-corrected for multiple comparisons), denoted by “1” in the last row and illustrated on the brain overlay in **(g)**.

Subsequently, we aggregated the spatial profiles of these inputs (i.e., *B* matrices) across participants for each consciousness state and employed PCA to discern the key patterns of task-induced activity. Notably, PCA uncovered a consistent PC across consciousness states, exhibiting highly comparable profiles. For example, Figures 4a–d illustrates PC3 based on cortical input profiles at different states—refer to Materials and Methods for detailed procedures. Notably, this component captures the activation of the auditory cortex in response to the auditory stimulus across all consciousness levels. These results suggest that the activation extends beyond the primary auditory cortex in the temporal lobe during awake and recovery states compared to sedation states. Furthermore, the awake and recovery states exhibit deactivation in the primary visual cortex relative to other states.

To pinpoint cortical regions undergoing the most pronounced changes across different consciousness levels within each participant, we isolated the column vector of *B* with the highest loading on PC3 for each subject and conducted an ANOVA test. The average identified *B* vector across participants is depicted in Figure 4f. These visualizations, along with Figure 4e, highlight the regions exhibiting significant differences across states (*p* < 0.05, FDR corrected). Noteworthy areas encompass various visual, somatomotor, limbic, and DMN regions.

**Figure 5:**
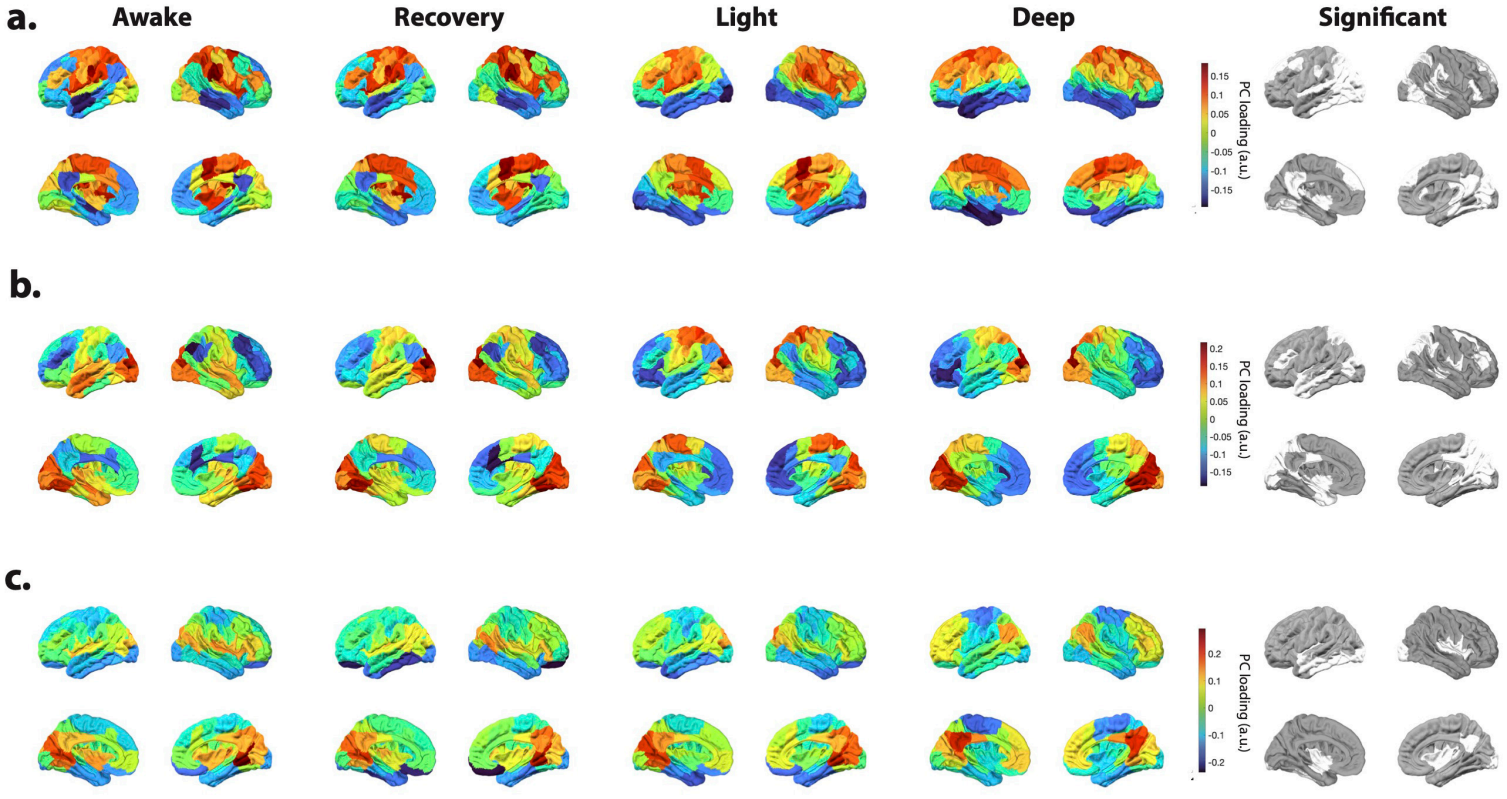
Principal component analysis (PCA) of the inputs' spatial profiles estimated during auditory stimulation paradigm. The first **(a)**, second **(b)**, and fourth **(c)** principal components of the inputs' spatial profiles, estimated during auditory stimulation. The last panel on the right shows regions with significant variations in the average inputs' spatial profiles (*B*) with the highest loading for each PC across consciousness states, as determined by ANOVA (*p* < 0.05, FDR corrected for multiple comparisons).

Additionally, Figure 5 depicts the outcomes of PCA conducted on the cortical *B* matrices, focusing on the three principal components (PC1, PC2, and PC4). These visualizations also emphasize the regions' significant differences in the average *B* vectors associated with each PC. Specifically, these PCs correspond to input patterns linked to the attention, somatomotor, and executive control networks (PC1), visual and attention (PC2), visual, executive control, and DMN (PC4). Note that while the estimated inputs' spatial profiles resemble previously identified transient co-activation patterns (CAPs) (Huang et al., 2020), their temporal profiles reveal a key difference. Unlike CAPs, these inputs allow for the presence of multiple input patterns at any given time point (Supplementary Figure 5).

Significantly, ANOVA results, akin to those for the auditory PC3, unveil state-dependent stimulus-induced changes (*p* < 0.05, FDR corrected). For instance, in PC2, visual inputs predominantly localize to occipital and parietal regions during deep sedation, whereas in the awake state, this pattern extends to temporal regions. Similarly, the PC4 pattern exhibits notable disparities in temporal and posterior cingulate cortex (PCC) regions contingent upon the level of consciousness, with more awake states showing a broader spread in the temporal lobe.

Moreover, we scrutinized the robustness of these findings concerning changes in model hyperparameters (i.e., input dimensions and regularization factor). The results, as illustrated in Supplementary Figure 6, demonstrate that the aforementioned observations are consistently captured even at higher input dimensions (*n* = 25 and λ = 0.5) and elevated regularization values (*n* = 10 and λ = 0.9). These collective findings underscore the efficacy of our framework in capturing task-induced patterns of large-scale network reconfigurations following alterations in consciousness levels.

**Figure 6:**
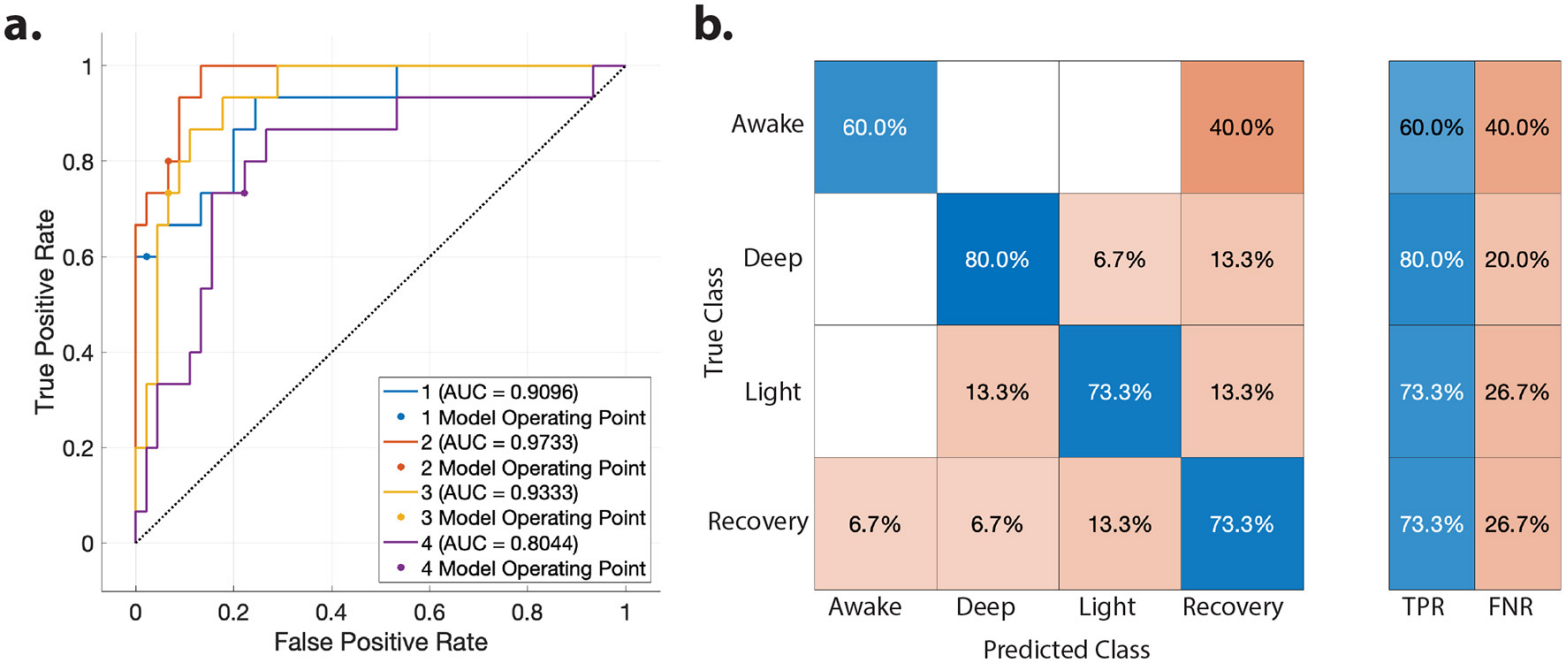
Classifying consciousness levels using spatial patterns of external inputs. **(a)** The Receiver Operating Characteristic (ROC) Curve for classification of different consciousness states with a linear SVM classifier using the vectors of input matrix *B* associated with PC1 through PC4 from Figures 4, 5. **(b)** Classification's confusion matrix, and true positive and false negative rates of each consciousness level.

Finally, to demonstrate the utility of the spatial input patterns unique to each consciousness level, we employed them to classify consciousness levels. Specifically, we utilized the vectors from the spatial input matrix *B* associated with the aforementioned PC1-4 across all subjects for consciousness level classification using a linear SVM classifier—see Materials and Methods for details. Our classification accuracy of 71.7% underscores the informative nature of the input patterns in tracking consciousness levels. We present the ROC and classification confusion matrix results in Figure 6.

## 4 Discussion

### 4.1 Resting-state dynamics for disorders of consciousness classification and closed-loop control

Resting-state paradigms offer a unique and powerful tool for studying consciousness due to their ability to assess brain activity independent of specific tasks. This advantage allows for direct comparisons across diverse populations, including healthy controls and patients with disorders of consciousness (DOC). Our approach focuses on characterizing the resting brain's spatiotemporal oscillatory patterns, enabling us to track how the level of consciousness modulates the stability and frequency of cortical networks. Our findings provide converging evidence that a hallmark of consciousness loss is the stabilization of oscillatory dynamics. This observation is supported by both electrophysiological data (Solovey et al., 2015; Alonso et al., 2014) and computational modeling studies (Sanz Perl et al., 2021; Piccinini et al., 2022).

Previous research has demonstrated a link between changes in the blood-oxygen-level-dependent (BOLD) signal frequency and alterations in consciousness state. For example, sleep can be characterized by changes in brain electrical activity, with NREM sleep exhibiting a shift toward low-frequency, high-amplitude EEG patterns. Building on this, recent studies employing simultaneous fMRI and EEG have identified distinct low-frequency and high-frequency BOLD oscillations in the brain during sleep transitions, each with unique spatiotemporal characteristics (Song et al., 2022). Our findings complement this work by revealing a significant decrease in the frequency of low-frequency modes encompassing the visual and somatomotor regions and, conversely, an increase in the frequency of high-frequency modes encompassing limbic regions as consciousness levels decline. Moreover, unlike traditional spectral analysis methods like the fast Fourier transform (FFT), which only reveals the temporal frequencies present in a system, our model's identified frequencies correspond to the eigenmodes of the system, capturing both the temporal and spatial nature of the oscillations.

The observation of unique changes in the spatiotemporal profile of the identified systems' dynamics has significant clinical implications. Firstly, by analyzing the spectral profiles of oscillatory modes, we can potentially extract informative features for patient classification within the multi-dimensional space of consciousness. This information could aid in predicting recovery for patients in difficult-to-classify states.

More importantly, our framework lays the groundwork for the development of novel therapeutic interventions. By estimating the dynamical system underlying brain activity, we can establish a mathematical objective function for the external control of brain oscillations. Specifically, with knowledge of the current and desired spectral profiles, we can leverage control theory to “steer” the pathological system toward healthy system dynamics. This could translate into designing targeted feedback stimulation protocols using electrical or transcranial stimulation techniques (Medaglia et al., 2017).

Future work should focus on applying this framework to DOC patients. By characterizing individual patient spectral profiles and their evolution during recovery, we can pave the way for closed-loop stimulation protocols. Ultimately, these protocols could be tested to evaluate the model's ability to promote recovery and transition patients from unconsciousness toward wakefulness.

### 4.2 Unveiling consciousness transitions through network reconfiguration dynamics and information integration

A significant advantage of our framework lies in its ability to bypass the need for prior knowledge about the external stimulus or the construction of hand-crafted features based on it. For instance, Kandeepan et al. (2020) relied on features extracted from the auditory stimulus, including time-domain properties (zero-crossing rate, energy) and frequency-domain properties (spectral centroid, Mel-frequency cepstral coefficients). While such features can identify activity patterns linked to external stimuli, they are limited to capturing low-level stimulus characteristics and cannot inherently capture the stimulus-induced activity on higher-level cognitive processes and activity. Additionally, anticipating relevant low-level features for different modalities can be challenging. As exemplified by Kandeepan et al. (2020), who employed 18 different features to identify potentially relevant ones, this approach can be cumbersome and potentially miss crucial information.

Our framework offers a significant advantage by revealing brain-wide network reconfigurations triggered by external stimuli. This goes beyond simply identifying changes in auditory processing areas, which is expected. Unlike conventional methods that rely on predefined ROIs or predetermined input regressors, our approach can uncover additional stimulus-related activity across various intrinsic brain networks by directly estimating the unknown external inputs driving brain activity. Therefore, it allows us to capture the combined effects of the stimulus's low-level features and its interaction with higher-level cognitive processes, providing a more comprehensive understanding of stimulus-induced brain dynamics.

Combining estimated system modes and external inputs sheds light on overlapping changes in network reconfigurations that might be missed by traditional methods like the general linear model (GLM) and FC analyses. For example, our results reveal that auditory stimulus-related input patterns spread beyond the primary auditory cortex in the temporal lobe, aligning with previous findings (Kandeepan et al., 2020). These results could indicate reduced complex and elaborated auditory processing in higher-order networks during sedated states. However, we also noted a significant deactivation of early visual areas in PC3 in the awake states, a finding not previously reported by Kandeepan et al. (2020). This suggests that the deactivation profile of visual areas may not be fully captured by the extracted features of the auditory stimulus.

Meanwhile, the PC2 analysis of input patterns reveals that during deep sedation, visual input patterns are confined to the occipital and parietal regions, whereas in wakefulness, they extend into higher-order auditory cortices. These findings indicate dynamic changes in network co-activation across different levels of consciousness. Moreover, the high consciousness level classification accuracy based on the input patterns highlights the consciousness-state-dependence of these activation/deactivation patterns. In wakefulness, auditory stimulation initially activates both primary and higher-order auditory areas, potentially leading to the deactivation of visual areas observed in PC3 patterns. However, the presence of an auditory-visual co-activation pattern in wakefulness (PC2) may also suggest visual processing of auditory information. Conversely, during deep and light sedation, there appears to be more segregated activation of primary sensory systems.

In addition to the auditory task-related co-activation patterns, we demonstrated that the estimated spatial input pattern captures various co-activation/deactivation patterns. In fact, the PCA analysis of these patterns uncovered that these patterns highly resemble the transient, momentary coactivation patterns (CAPs) described in several previous studies (Liu et al., 2018, 2013; Liu and Duyn, 2013; Huang et al., 2020). For instance, PC1, PC2, and PC4 are similar to the DAT+/(DMN-), VIS+/(VAT-), DMN+/(DAT-) CAP identified in Huang et al. (2020). However, the primary distinction between CAPs and the identified inputs in the LTI system is that, unlike CAPs, multiple input patterns can coexist simultaneously at any given time point.

Interpreting these input patterns requires careful consideration, particularly with respect to whether they reflect purely exogenous drivers or stimulus-triggered modulations of intrinsic dynamical modes. Within the LTI framework, the system matrix *A* captures autonomous oscillatory dynamics, while estimated inputs represent deviations from this baseline that cannot be explained by intrinsic evolution alone. Biologically, naturalistic auditory stimuli trigger cascading responses across networks via thalamocortical circuits and higher-order thalamic nuclei, facilitating information propagation beyond primary sensory cortices (Hwang et al., 2017; Shine, 2021). The observed consciousness-dependent spatial reorganization likely reflects anesthetic-induced alterations in neuromodulatory tone and synaptic gain, which fundamentally restrict the repertoire of functional network configurations and disrupt input propagation through cortical hierarchies (Brown et al., 2011; Mashour and Hudetz, 2018). For instance, the awake-state recruitment of visual, attention, and executive networks during auditory processing may reflect top-down predictive processes and cross-modal binding (Lerner et al., 2011). Consequently, these patterns are best conceptualized as stimulus-induced modulations of intrinsic dynamics, capturing how external perturbations drive consciousness-dependent shifts in the brain's global response modes.

Overall, our framework offers a more nuanced understanding of how information integrates across modalities and intrinsic brain systems during different consciousness states. This has significant clinical implications, particularly for DOC patients. By combining complex naturalistic stimuli with the input patterns identified by our model, we can potentially develop more objective and reliable methods for assessing awareness in DOC patients. This approach could involve examining how information is processed and shared across sensory and higher-level associative brain networks. This could lead to improved diagnosis and prognosis and, ultimately, the development of targeted interventions for DOC patients.

Our framework is closely related to a growing class of machine learning approaches that model system dynamics and responses to perturbations rather than relying on static representations. In AI, recurrent neural networks (Sussillo and Abbott, 2009), reservoir computing systems (Jaeger and Haas, 2004), and other dynamical-systems-based models are commonly analyzed through their stability properties and responses to external perturbations, with transitions near stability boundaries or critical regimes linked to enhanced computational capacity, adaptability, and input sensitivity (Bertschinger et al., 2004). Recent work has further emphasized that such responsiveness reflects non-equilibrium dynamics, with functional regimes characterized by broken detailed balance and state-dependent stability (Nartallo-Kaluarachchi et al., 2025). Similarly, our approach characterizes large-scale brain dynamics through the stability and oscillatory structure of intrinsic modes and aligns with findings from both artificial and biological systems, showing that proximity to criticality and heightened response functions accompany transitions between functional regimes (Du and Huang, 2025; Sooter et al., 2025). What distinguishes our approach is its explicit treatment of unknown external inputs. Many existing frameworks either assume autonomous dynamics or implicitly treat inputs, whereas stochastic criticality-based models primarily emphasize regime identification without explicitly modeling how time-varying external drives reshape system dynamics. By explicitly modeling the interaction between intrinsic dynamics and unknown inputs, our input–output formulation provides a complementary and tractable framework for jointly characterizing intrinsic stability structure and externally driven modulation.

### 4.3 Limitations and future directions

This study has several limitations that motivate future research directions. First, we utilized a publicly available dataset with a limited scan duration per subject. While we mitigated this by estimating a single group-level system matrix to capture slow brain oscillations, ideally, future studies should employ longer resting-state scans (15–30 min) for each participant to enable subject-specific system models, potentially leading to more accurate predictions.

Second, ventral brain regions generally exhibit lower signal-to-noise ratios (SNR) (Rua et al., 2018). To address this, we excluded participants with missing ROI data and lowered the threshold for some brain voxels to include these regions. This approach might have introduced additional noise, and future work should utilize datasets specifically designed to enhance SNR for a more reliable assessment of these regions.

Another limitation of this study is the modest sample size, which may constrain statistical power and generalizability. Future work will address this by leveraging resampling-based approaches to assess the stability of the observed state-dependent differences in eigenmode properties and by validating these findings in larger, independent datasets.

Finally, our framework adopts a linear and time-invariant description of large-scale brain dynamics. This modeling choice does not imply that brain dynamics are intrinsically linear or stationary, but rather reflects evidence that, at macroscopic spatial scales and short predictive horizons, linear models provide statistically optimal and interpretable descriptions of observed neural activity (Nozari et al., 2024). Such apparent linearity can arise from spatiotemporal averaging, observation noise, and limited data length, even when underlying microscale dynamics are nonlinear (Nozari et al., 2024). Importantly, this does not preclude the relevance of time-varying or nonlinear models, which are likely essential for capturing longer-term dynamics, state transitions, and metastability (Nartallo-Kaluarachchi et al., 2025). In this context, our LTI framework with unknown inputs serves a complementary role, providing a parsimonious baseline that isolates where linear dynamics fail, allowing residual inputs to reflect a mixture of unmodeled nonlinear interactions and external or neuromodulatory drivers. While it remains challenging to distinguish a time-invariant system driven by time-varying inputs from a truly time-varying system with nonlinearities, future work could explicitly compare these modeling regimes. Such comparisons help clarify when non-stationary or nonlinear formulations provide additional explanatory power, particularly for understanding transitions in consciousness and forecasting recovery trajectories in disorders of consciousness.

## 5 Conclusions

This study sheds light on the neural underpinnings of consciousness by analyzing the interplay between spatiotemporal oscillatory patterns and their external drivers within large-scale brain networks. We demonstrate a critical link between consciousness levels and the dynamics of these networks, with a shift toward stabilized oscillations characterizing unconsciousness. Importantly, our framework offers an principled, data-driven approach to studying consciousness, bypassing the need for predefined features and revealing consciousness-level-dependent brain-wide reconfigurations of external drivers of brain dynamics. Significantly impacting clinical care, our research can guide the development of objective assessment tools and targeted interventions for disorders of consciousness.

## Data Availability

The original contributions presented in the study are included in the article/Supplementary material, further inquiries can be directed to the corresponding author.
