# Supplementary material for: Shifts in brain dynamics and drivers of consciousness state transitions
Source: Front Comput Neurosci. 2026 Feb 10;20:1731868. doi: 10.3389/fncom.2026.1731868 (PMC12929524; doi:10.3389/fncom.2026.1731868)
Supplement: Supplementary file 1 [file Data_Sheet_1.pdf]

frontiers

Supporting information for ”Shifts in Brain Dynamics and Drivers of Consciousness State Transitions”

1 JOINT-ESTIMATION ALGORITHM.

In what follows, we seek to determine the dynamics and input matrices, as well as the input sequence of the following dynamical system,

$$x[k+1] = Ax[k] + Bu[k], \tag{S1}$$

where $x[k] \in \mathbb{R}^n$ denotes the state, $u[k] \in \mathbb{R}^p$ denotes the input, $A \in \mathbb{R}^{n\times n}$ the dynamics matrix, and $B \in \mathbb{R}^{n\times p}$ the input matrix. Specifically, by considering a sequence of data $\{z[k]\}_{k=0}^{N-1}$, we seek to find an approximation of the parameters $(A, B, \{u[k]\}_{k=0}^{N-1})$ denoted by $(\tilde{A}, \tilde{B}, \{\tilde{u}[k]\}_{k=0}^{N-1})$, respectively. Next, notice that given the dynamical system (S9), we obtain

$$z[k] = Az[k-1] + Bu[k-1] + \varepsilon_k, \tag{S2}$$

where the error can be captured by $\varepsilon_k \sim \mathcal{N}(0, \Sigma)$. Subsequently, in a least-squares minimization sense, we seek to minimize the following

$$\min_{(\tilde{A},\tilde{B},\{\tilde{u}[k]\}_{k=0}^{N-1})} \sum_{k=1}^{N} \|z[k] - \left(\tilde{A}z[k-1] + \tilde{B}\tilde{u}[k-1]\right)\|_2^2, \tag{S3}$$

Additionally, since both the input matrix and sequence of inputs are unknown, the problem is not well posed, which forces us to consider possible feasibility constraints (e.g., $\|\tilde{u}[k]\| \leq 1$) and the objective should be changed to account for a regularization term (e.g., the 1-norm) that penalize the number of non-zero entries, which lead to the following problem.

$$\min_{(\tilde{A},\tilde{B},\{\tilde{u}[k]\}_{k=0}^{N-1})} \sum_{k=1}^{N} \|z[k] - \left(\tilde{A}z[k-1] + \tilde{B}\tilde{u}[k-1]\right)\|_2^2 + \lambda\|\tilde{u}[k-1]\|_1^2, \tag{S4}$$

where $\lambda \in \mathbb{R}_0^+$ is the regularization term that weights the tradeoffs between the approximation error and the number of nonzero entries. To determine the unknown parameters $(\tilde{A}, \tilde{B}, \{\tilde{u}[k]\}_{k=0}^{N-1})$ that is the solution to the above problem, we will consider an algorithm that borrows ideas from the Expectation-Maximization algorithm, which details can be found in Gupta et al. (2019, 2018). Briefly, we seek to determine a converging sequence of unknown parameters $(\tilde{A}^{(l)}, \tilde{B}^{(l)}, \{\tilde{u}^{(l)}[k]\}_{k=0}^{N-1})$, which initial parameters (i.e., with $l = 0$) are set as follows: (*i*) $A^{(0)}$ is the solution to (S11) with input matrix and inputs set to zero; and (ii) $\tilde{B}^{(0)}$ is initialized at random which entries are obtained from standard Gaussians. Next, for a predefined value of λ in (S12), we find a solution to it by proceeding sequentially as follows (for each iteration $l = 1, 2, \ldots$):

noindent (*i*) determine $\{\tilde{u}^{(l)}[k]\}_{k=0}^{N-1}$, given the values $(\tilde{A}^{(l-1)}, \tilde{B}^{(l-1)})$

$$\min_{\{\tilde{u}^{(l)}[k]\}_{k=0}^{N-1}} \sum_{k=1}^{N} \|z[k] - \left(\tilde{A}^{(l-1)} z[k-1] + \tilde{B}^{(l-1)} \tilde{u}^{(l)}[k-1]\right) \|_2^2 + \lambda \|\tilde{u}^{(l)}[k-1]\|_1^2, \tag{S5}$$

(*ii*) determine $\tilde{A}^{(l)}$ given the values $(\tilde{B}^{(l-1)}, \{\tilde{u}^{(l)}[k]\}_{k=0}^{N-1})$, which is the solution to the following problem

$$\min_{(\tilde{A}^{(l)})} \sum_{k=1}^{N} \|z[k] - \left(\tilde{A}^{(l)} z[k-1] + \tilde{B}^{(l-1)} \tilde{u}^{(l)}[k-1]\right) \|_2^2 + \lambda \|\tilde{u}^{(l)}[k-1]\|_1^2, \tag{S6}$$

(*iii*) determine $\tilde{B}^{(l)}$ given the values $(\tilde{A}^{(l)}, \{\tilde{u}^{(l)}[k]\}_{k=0}^{N-1})$, which is the solution to the following problem

$$\min_{(\tilde{B}^{(l)})} \sum_{k=1}^{N} \|z[k] - \left(\tilde{A}^{(l)} z[k-1] + \tilde{B}^{(l)} \tilde{u}^{(l)}[k-1]\right) \|_2^2 + \lambda \|\tilde{u}^{(l)}[k-1]\|_1^2, \tag{S7}$$

and (*iv*) determine $\tilde{A}^{(l)}$ given the values $(\tilde{B}^{(l)}, \{\tilde{u}^{(l)}[k]\}_{k=0}^{N-1})$, which is the solution to the following problem

$$\min_{(\tilde{A}^{(l)})} \sum_{k=1}^{N} \|z[k] - \left(\tilde{A}^{(l)} z[k-1] + \tilde{B}^{(l)} \tilde{u}^{(l)}[k-1]\right) \|_2^2 + \lambda \|\tilde{u}^{(l)}[k-1]\|_1^2. \tag{S8}$$

Lastly, it is important to notice that several criteria can be adopted to stop the sequential procedure in the algorithm. In particular, we have considered the total variation between iterations on the inputs to be below a threshold.

1 JOINT-ESTIMATION ALGORITHM.

In what follows, we seek to determine the dynamics and input matrices, as well as the input sequence of the following dynamical system,

$$x[k+1] = Ax[k] + Bu[k], \tag{S9}$$

where $x[k] \in \mathbb{R}^n$ denotes the state, $u[k] \in \mathbb{R}^p$ denotes the input, $A \in \mathbb{R}^{n\times n}$ the dynamics matrix, and $B \in \mathbb{R}^{n\times p}$ the input matrix. Specifically, by considering a sequence of data $\{z[k]\}_{k=0}^{N-1}$, we seek to find an approximation of the parameters $(A, B, \{u[k]\}_{k=0}^{N-1})$ denoted by $(\tilde{A}, \tilde{B}, \{\tilde{u}[k]\}_{k=0}^{N-1})$, respectively. Next, notice that given the dynamical system (S9), we obtain

$$z[k] = Az[k-1] + Bu[k-1] + \varepsilon_k, \tag{S10}$$

where the error can be captured by $\varepsilon_k \sim \mathcal{N}(0, \Sigma)$. Subsequently, in a least-squares minimization sense, we seek to minimize the following

$$\min_{(\tilde{A},\tilde{B},\{\tilde{u}[k]\}_{k=0}^{N-1})} \sum_{k=1}^{N} \|z[k] - \left(\tilde{A}z[k-1] + \tilde{B}\tilde{u}[k-1]\right)\|_2^2, \tag{S11}$$

Additionally, since both the input matrix and sequence of inputs are unknown, the problem is not well posed, which forces us to consider possible feasibility constraints (e.g., $\|\tilde{u}[k]\| \leq 1$) and the objective should be changed to account for a regularization term (e.g., the 1-norm) that penalize the number of non-zero entries, which lead to the following problem.

$$\min_{(\tilde{A},\tilde{B},\{\tilde{u}[k]\}_{k=0}^{N-1})} \sum_{k=1}^{N} \|z[k] - \left(\tilde{A}z[k-1] + \tilde{B}\tilde{u}[k-1]\right)\|_2^2 + \lambda\|\tilde{u}[k-1]\|_1^2, \tag{S12}$$

where $\lambda \in \mathbb{R}_0^+$ is the regularization term that weights the tradeoffs between the approximation error and the number of nonzero entries. To determine the unknown parameters $(\tilde{A}, \tilde{B}, \{\tilde{u}[k]\}_{k=0}^{N-1})$ that is the solution to the above problem, we will consider an algorithm that borrows ideas from the Expectation-Maximization algorithm, which details can be found in Gupta et al. (2019, 2018). Briefly, we seek to determine a converging sequence of unknown parameters $(\tilde{A}^{(l)}, \tilde{B}^{(l)}, \{\tilde{u}^{(l)}[k]\}_{k=0}^{N-1})$, which initial parameters (i.e., with $l = 0$) are set as follows: (*i*) $A^{(0)}$ is the solution to (S11) with input matrix and inputs set to zero; and (ii) $\tilde{B}^{(0)}$ is initialized at random which entries are obtained from standard Gaussians. Next, for a predefined value of λ in (S12), we find a solution to it by proceeding sequentially as follows (for each iteration $l = 1, 2, \ldots$):

noindent (*i*) determine $\{\tilde{u}^{(l)}[k]\}_{k=0}^{N-1}$, given the values $(\tilde{A}^{(l-1)}, \tilde{B}^{(l-1)})$

$$\min_{\{\tilde{u}^{(l)}[k]\}_{k=0}^{N-1}} \sum_{k=1}^{N} \|z[k] - \left(\tilde{A}^{(l-1)}z[k-1] + \tilde{B}^{(l-1)}\tilde{u}^{(l)}[k-1]\right)\|_2^2 + \lambda\|\tilde{u}^{(l)}[k-1]\|_1^2, \tag{S13}$$

(*ii*) determine $\tilde{A}^{(l)}$ given the values $(\tilde{B}^{(l-1)}, \{\tilde{u}^{(l)}[k]\}_{k=0}^{N-1})$, which is the solution to the following problem

$$\min_{(\tilde{A}^{(l)})} \sum_{k=1}^{N} \|z[k] - \left(\tilde{A}^{(l)} z[k-1] + \tilde{B}^{(l-1)} \tilde{u}^{(l)}[k-1]\right) \|_2^2 + \lambda \|\tilde{u}^{(l)}[k-1]\|_1^2, \tag{S14}$$

(*iii*) determine $\tilde{B}^{(l)}$ given the values $(\tilde{A}^{(l)}, \{\tilde{u}^{(l)}[k]\}_{k=0}^{N-1})$, which is the solution to the following problem

$$\min_{(\tilde{B}^{(l)})} \sum_{k=1}^{N} \|z[k] - \left(\tilde{A}^{(l)} z[k-1] + \tilde{B}^{(l)} \tilde{u}^{(l)}[k-1]\right) \|_2^2 + \lambda \|\tilde{u}^{(l)}[k-1]\|_1^2, \tag{S15}$$

and (*iv*) determine $\tilde{A}^{(l)}$ given the values $(\tilde{B}^{(l)}, \{\tilde{u}^{(l)}[k]\}_{k=0}^{N-1})$, which is the solution to the following problem

$$\min_{(\tilde{A}^{(l)})} \sum_{k=1}^{N} \|z[k] - \left(\tilde{A}^{(l)} z[k-1] + \tilde{B}^{(l)} \tilde{u}^{(l)}[k-1]\right) \|_2^2 + \lambda \|\tilde{u}^{(l)}[k-1]\|_1^2. \tag{S16}$$

Lastly, it is important to notice that several criteria can be adopted to stop the sequential procedure in the algorithm. In particular, we have considered the total variation between iterations on the inputs to be below a threshold.

2 DETERMINING THE OPTIMAL NUMBER OF EIGENVECTOR CLUSTERS.

To find the optimal number of eigenvector clusters, we used the following methods.

Elbow Method

K-means clustering aims to minimize the within-cluster variance or the within-cluster sum of squared errors (SSE). Ideally, this error should be as small as possible. However, increasing the number of clusters always reduces the SSE. The Elbow method involves plotting SSE as a function of the number of clusters and identifying a point (the ”elbow”) where adding another cluster does not significantly reduce the SSE. This elbow typically indicates the optimal number of clusters, although it may not always be easily identifiable, especially in less clustered data.

Calinski-Harabasz Criterion

We used the Calinski-Harabasz criterion (Caliński and Harabasz, 1974), also known as the variance ratio criterion, defined as:

$$VRC = \frac{SS_B}{SS_W} \times \frac{(N-k)}{(k-1)}, \tag{S17}$$

where k is the number of clusters, N is the number of observations, and SS_B and SS_W are the total between-cluster and within-cluster variance, respectively. SS_B and SS_W are defined as:

$$SS_B = \sum_{i=1}^{k} n_i \left\| m_i - m \right\|^2, \tag{S18}$$

$$SS_W = \sum_{i=1}^{k} \sum_{x \in c_i} \left\| x - m_i \right\|^2, \tag{S19}$$

where n_i is the number of points in the ith cluster, m_i is the centroid of the ith cluster, m is the overall mean of the data, and $\|\cdot\|$ denotes the Euclidean distance. The optimal number of clusters maximizes the variance ratio criterion, indicating well-defined clusters with high between-cluster variance and low within-cluster variance.

Davies-Bouldin Criterion

The Davies-Bouldin criterion (Davies and Bouldin, 1979) measures the ratio of within-cluster to between-cluster distances and is defined as:

$$DB = \frac{1}{k} \sum_{i=1}^{k} \max_{j \neq i} D_{i,j}, \tag{S20}$$

where $D_{i,j}$ is the ratio of within-cluster to between-cluster distance for clusters i and j, defined as:

$$D_{i,j} = \frac{\bar{d}_i + \bar{d}_j}{d_{i,j}}, \tag{S21}$$

where $\bar{d}_i$ and $\bar{d}_j$ are the average distances of data points in clusters i and j to their respective centroids, and $d_{i,j}$ is the Euclidean distance between the centroids of clusters i and j. The optimal number of clusters minimizes the Davies-Bouldin index, representing the best within-to-between cluster distance ratio.

Silhouette Criterion

The Silhouette criterion (Rousseeuw, 1987) measures how similar each point is to its own cluster compared to other clusters and is defined as:

$$S_i = \frac{b_i - a_i}{\max(a_i, b_i)}, \tag{S22}$$

where a_i is the average distance between the ith data point and other points in its cluster, and b_i is the minimum average distance between the ith data point and points in different clusters. A high Silhouette score (ranging between 1 and -1) indicates that the data point is well-clustered within its own cluster and poorly matches data points from other clusters. Conversely, many data points with zero or negative Silhouette values suggest the presence of too few or too many clusters.

REFERENCES

Caliński, T. and Harabasz, J. (1974). Communications in Statistics - Theory and Methods. *Communications in Statistics* 3, 1–27. doi:10.1080/03610927408827101

Davies, D. L. and Bouldin, D. W. (1979). A Cluster Separation Measure. *IEEE Transactions on Pattern Analysis and Machine Intelligence* PAMI-1, 224–227. doi:10.1109/TPAMI.1979.4766909

Gupta, G., Pequito, S., and Bogdan, P. (2018). Dealing with unknown unknowns: Identification and selection of minimal sensing for fractional dynamics with unknown inputs. In *2018 Annual American Control Conference (ACC)* (IEEE), 2814–2820

Gupta, G., Pequito, S., and Bogdan, P. (2019). Learning latent fractional dynamics with unknown unknowns. *Proceedings of the American Control Conference* 2019-July, 217–222. doi:10.23919/acc.2019.8815074

Rousseeuw, P. J. (1987). Silhouettes: A graphical aid to the interpretation and validation of cluster analysis. *Journal of Computational and Applied Mathematics* 20, 53–65. doi:10.1016/0377-0427(87)90125-7

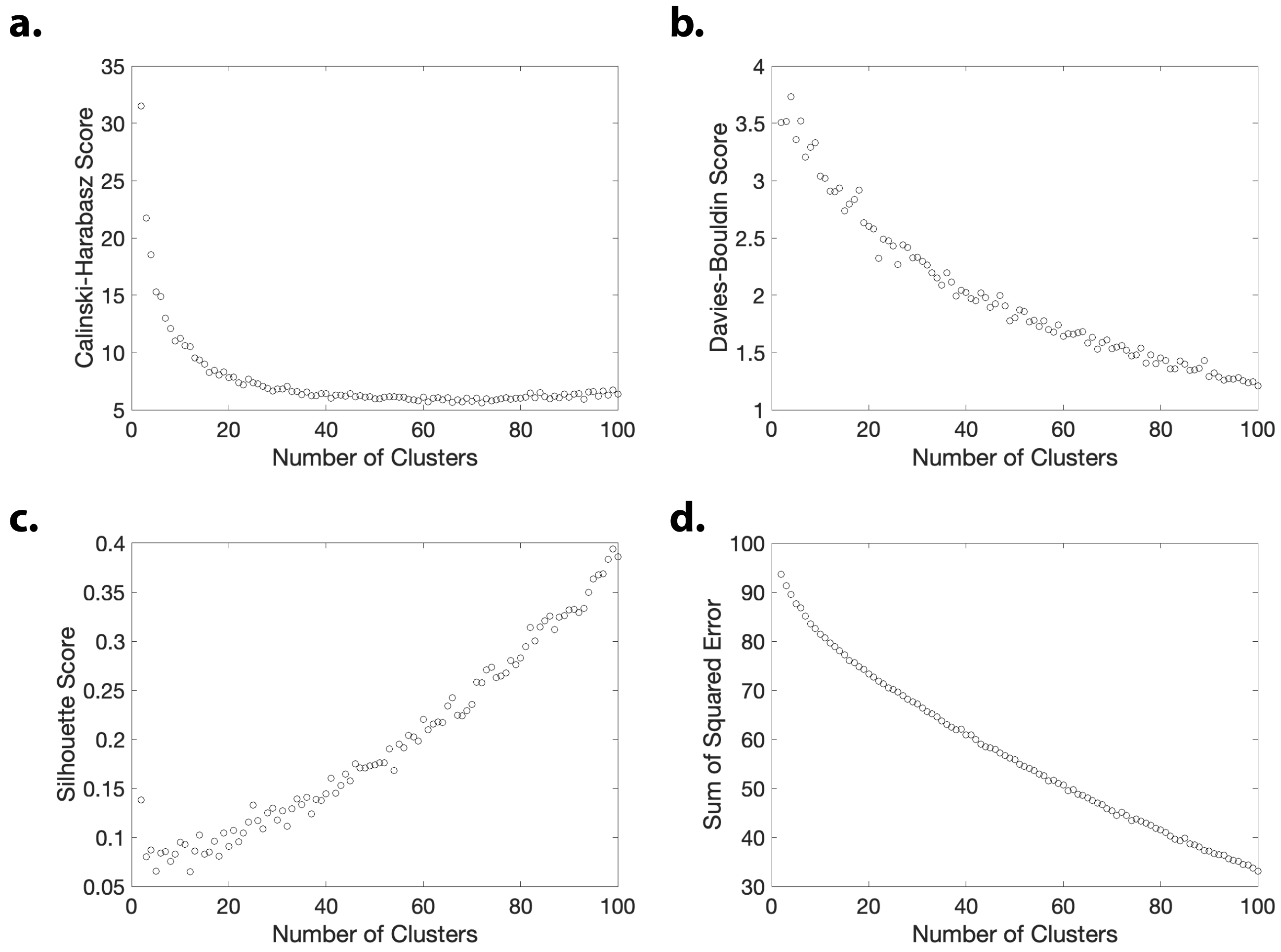

Figure S1. Determining the Number of Eigenvector Clusters. To evaluate the optimal number of clusters (k) present in the eigenvectors, we used the ***a.*** CalinskiHarabasz criterion (ratio of within-cluster to between-cluster dispersion) (Caliński and Harabasz, 1974), ***b.*** Davies-Bouldin criterion (ratio of within-cluster to between-cluster distances) (Davies and Bouldin, 1979), and ***c.*** Silhouette criterion (measure of similarity of each data point to other points in its cluster compared to points in other clusters) (Rousseeuw, 1987). Based on these three criteria, the optimal number of clusters in the data is $k = 2, 100$, and 99, respectively, as shown in panels **a-c**. ***d.*** The plot represents the sum of squared errors (distances to cluster centers). Due to the smoothness of the curve, a single k corresponding to the *'elbow'* of the curve in panel ***d*** cannot be identified. Together, these results suggest that the data may not contain clearly defined clusters for $k > 2$. Therefore, to examine the organization of eigenvectors at higher resolutions in the main manuscript, we examined $k = 3 - 20$, which roughly corresponds to the elbow of the curve.

3 SUPPLEMENTARY FIGURES.

- Eigenvalue Angle (θ)

Cluster #	df1	df2	F	p	η^2	Cohen f
1	2	4	0.6129	0.5859	0.2346	0.5536
2	3	29	0.9609	0.4244	0.0904	0.3153
3	3	34	0.8700	0.4661	0.0713	0.2771
4	3	46	1.6403	0.1931	0.0966	0.3271
5	3	94	3.3849	0.0214	0.0975	0.3287
6	3	83	6.2531	7.0254e-04	0.1844	0.4754
7	3	39	12.5484	6.9213e-06	0.4912	0.9825
8	3	40	1.9271	0.1407	0.1263	0.3802

- Eigenvalue Stability (λ)

Cluster #	df1	df2	F	p	η^2	Cohen f
1	2	4	5.5455	0.0703	0.7349	1.6652
2	3	29	0.6099	0.6140	0.0593	0.2512
3	3	34	2.6239	0.0663	0.1880	0.4812
4	3	46	1.1025	0.3577	0.0671	0.2681
5	3	94	33.0183	1.1515e-14	0.5131	1.0265
6	3	83	7.3512	1.9847e-04	0.2099	0.5155
7	3	39	23.3692	7.9985e-09	0.6426	1.3408
8	3	40	1.2710	0.2973	0.0870	0.3087

Figure S2. Effect sizes and significance tests for cluster-wise spectral differences. ANOVA results testing for significant differences in eigenmode stability ($|\lambda|$) and frequency (θ) across the four consciousness states for each k-means cluster presented in Fig. 3. For each cluster, the table reports: degrees of freedom between groups (df1) and within groups (df2), F-statistic, p-value, partial eta-squared (η^2) as a measure of effect size, and Cohen's f. Clusters 5 and 7 showing significant state-dependent changes in both stability and frequency which correspond to those highlighted with '*' and 'o' markers in Fig. 1.

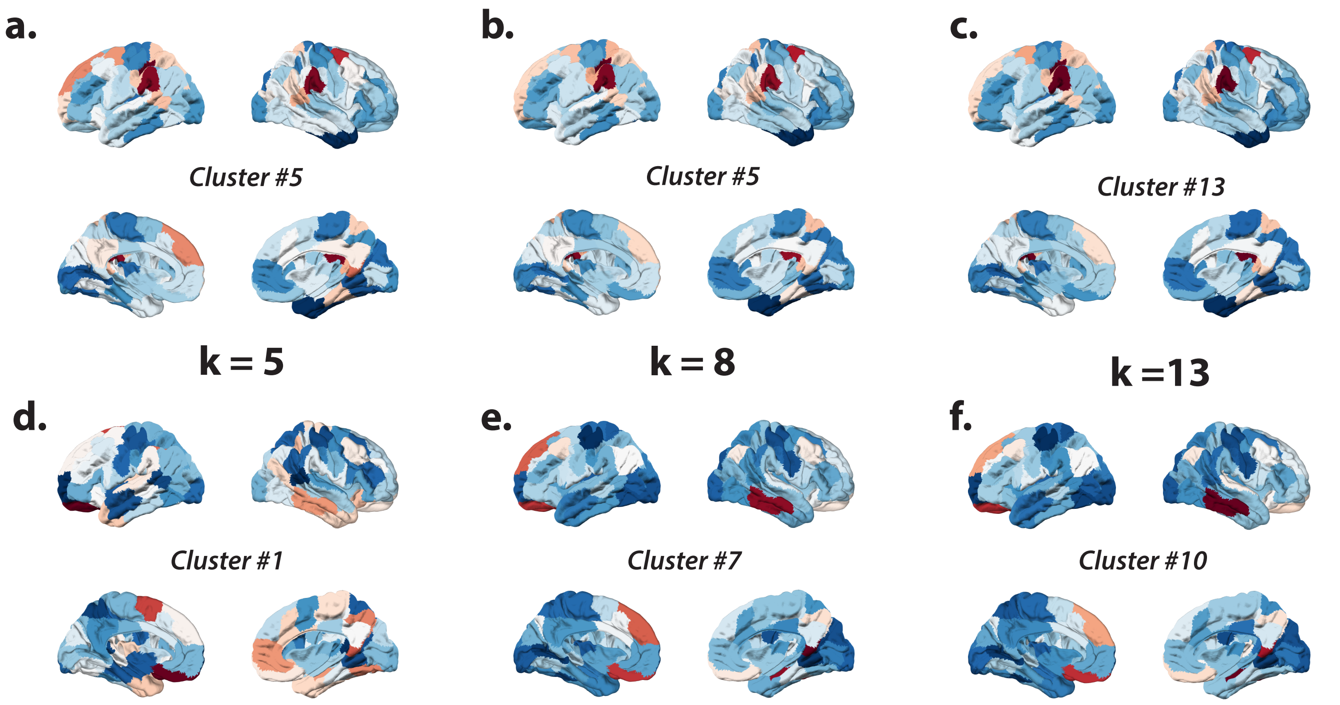

Figure S3. Robust identification of eigenmodes showing altered spectral profiles during sedation-induced loss of consciousness. Mean cluster centroids for the two clusters exhibiting state-dependent effects (marked with '*' (Panels **a-c**) and 'o' (Panels **d-f**) in Fig. 1a-b shown across three clustering resolutions (k = 5, 8, and 13). Spatial patterns demonstrate high consistency across different k values, indicating robust identification of these functionally relevant eigenmodes despite changes in clustering resolution.

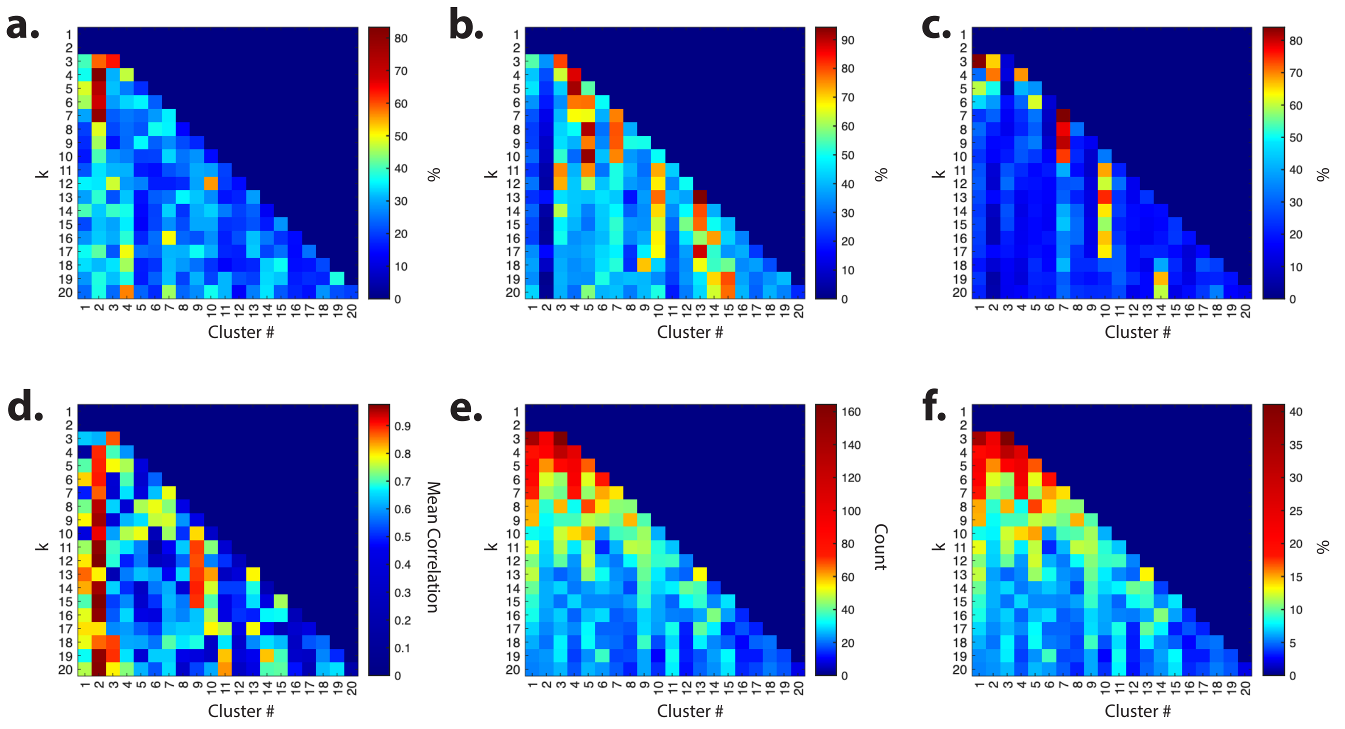

Figure S4. Stability and replicability of identified eigenmodes across clustering iterations. a. Percentage of variance explained by the first principal component applied to all centroids associated with each cluster across 100,000 k-means iterations. Higher values indicate stable and repeatable cluster identification across iterations. **b.** Percentage of iterations (out of 100,000 repetitions) in which ANOVA revealed significant differences ($p < 0.05$, FDR-corrected for multiple comparisons) in eigenmode stability ($|\lambda|$) across consciousness states for each cluster. Cluster centroids are sorted from smallest to largest k using the Hungarian algorithm (see Methods). **c.** Same as panel **b**, but for eigenmode frequency (angle) instead of stability. **d.** Pearson correlation between each identified cluster centroid and the reference centroid from the first iteration (used for Hungarian sorting across all iterations). Higher values indicate stable spatial patterns across repetitions. **e.** Mean number of eigenmodes assigned to each cluster across iterations. **f.** Percentage of total eigenmodes assigned to each cluster (normalized version of panel **e**), illustrating the relative size of each cluster.

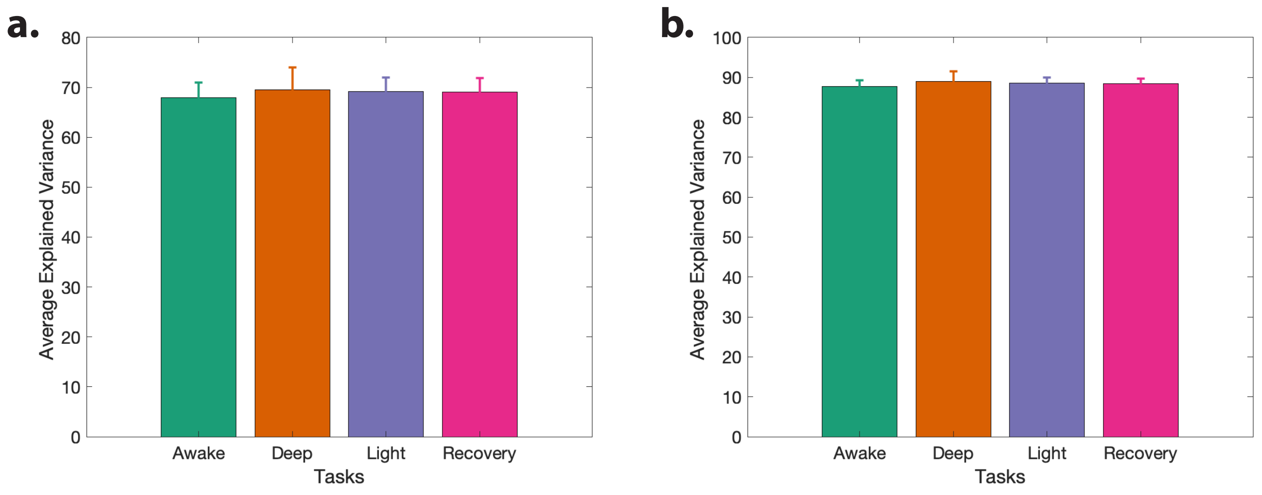

Figure S5. Principal Component Analysis (PCA) of the LTI model's residuals during auditory stimulation paradigm. The mean and standard deviation (error bars) of the explained variance using the first 10 (**a**) and 25 (**b**) PCs calculated from the residuals of the LTI model using auditory stimulation scans across different consciousness levels. The average mean explained variance across all consciousness levels is 68.9% and 88.4% in panels **a** and **b**, respectively.

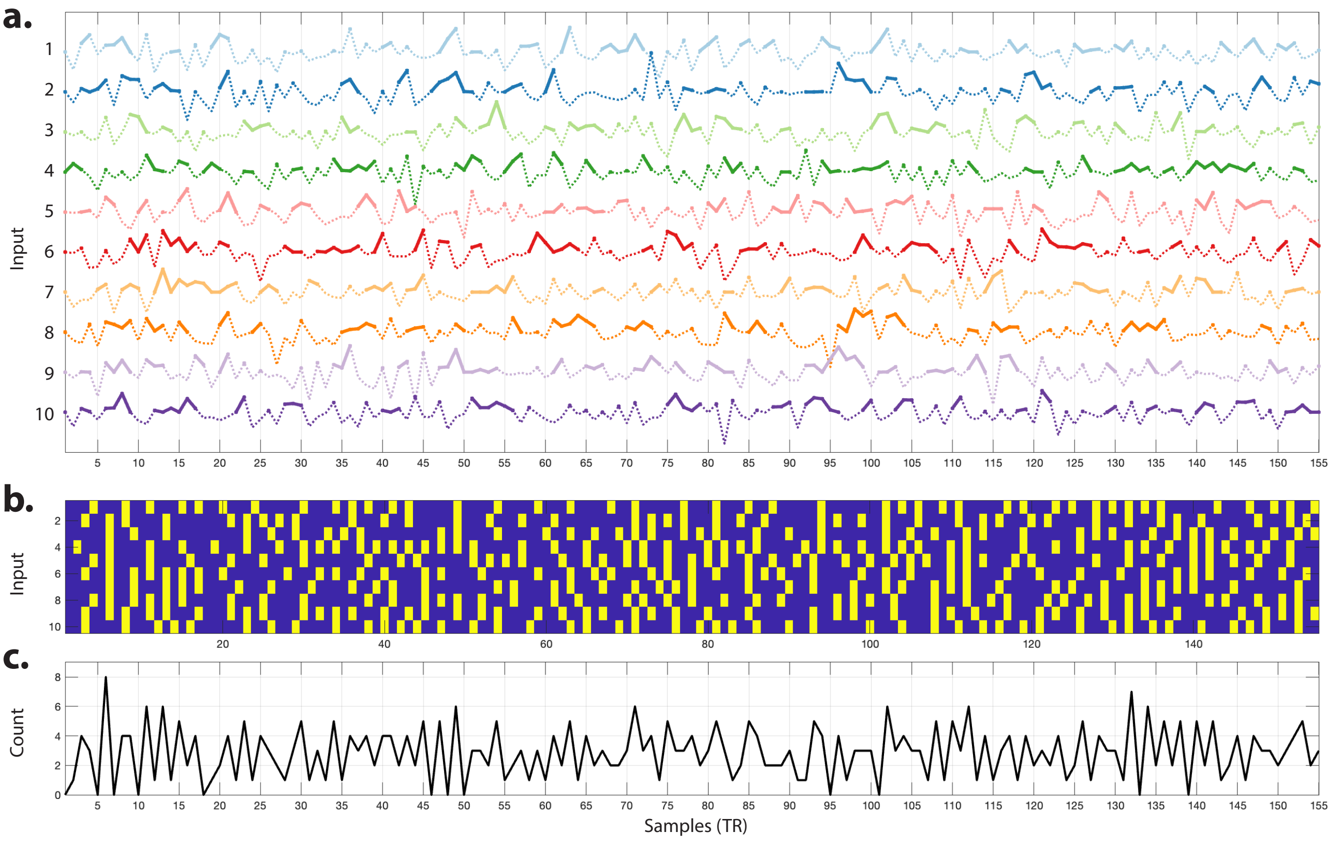

Figure S6. Sample subject's estimated inputs' temporal profiles. (**a**) Color-coded time series shows the temporal profile of the ten inputs during the auditory simulation task in a sample subject during wakeful states. The dashed lines highlight the time points below the inputs' mean. (**b**) local peaks with above mean values in panel **a** time series. (**c**) Total number of identified peaks at each time point in panel **b**.

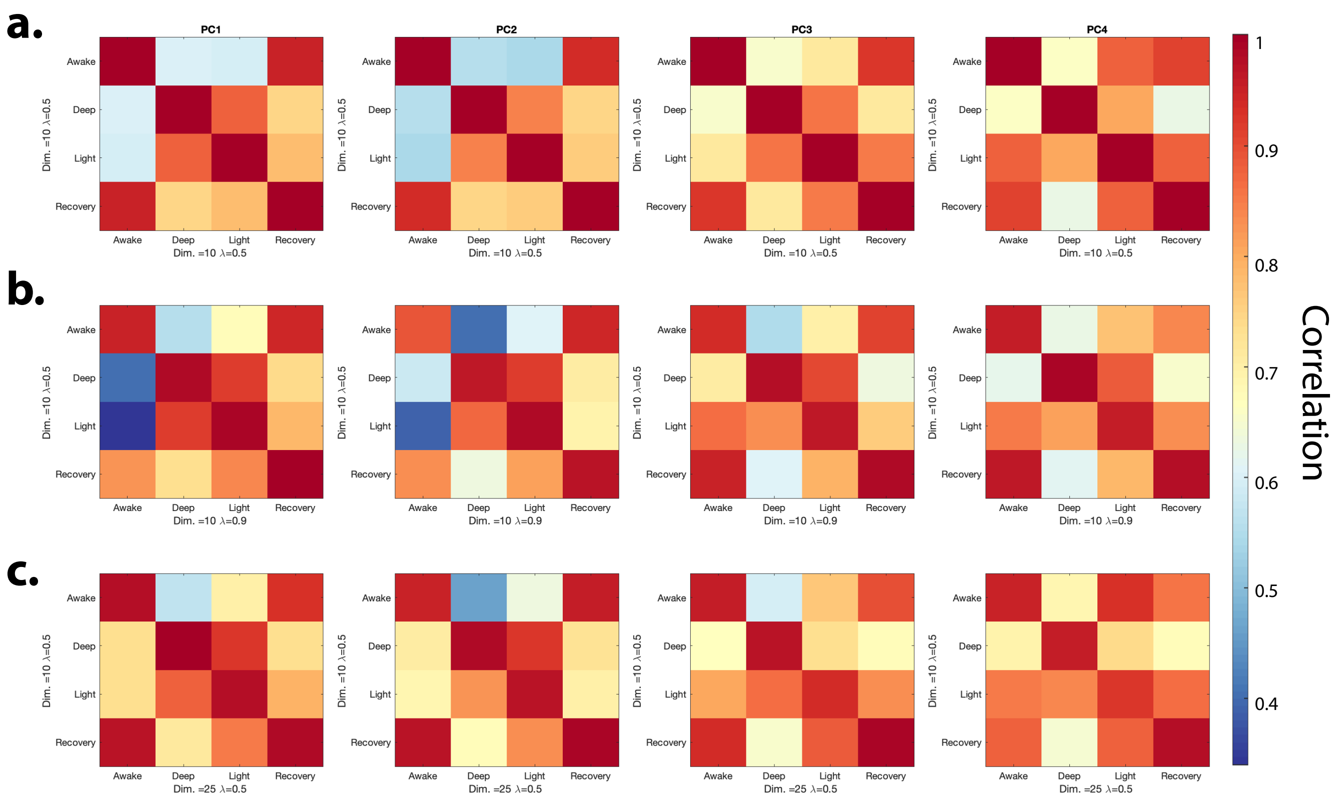

Figure S7. Comparison of leading input Principal Components (PCs) across hyperparameters This figure compares the leading principal components (PCs 1-4) identified from the spatial profiles of estimated inputs (matrix B) across different consciousness states. The analysis explores the impact of varying hyperparameters on the extracted PCs.**(a)** Correlation matrix showing the similarity between PCs identified using an input dimension of 10 and a regularization factor of 0.5. **(b)** Correlation matrix showing the similarity between PCs identified using an input dimension of 10 and a regularization factor of 0.5 (same as panel **a**), compared to those identified using an input dimension of 10 and a regularization factor of 0.9. **(c)** Correlation matrix showing the similarity between PCs identified using an input dimension of 10 and a regularization factor of 0.5 (same as panel **a**), compared to those identified using an input dimension of 25 and a regularization factor of 0.5.

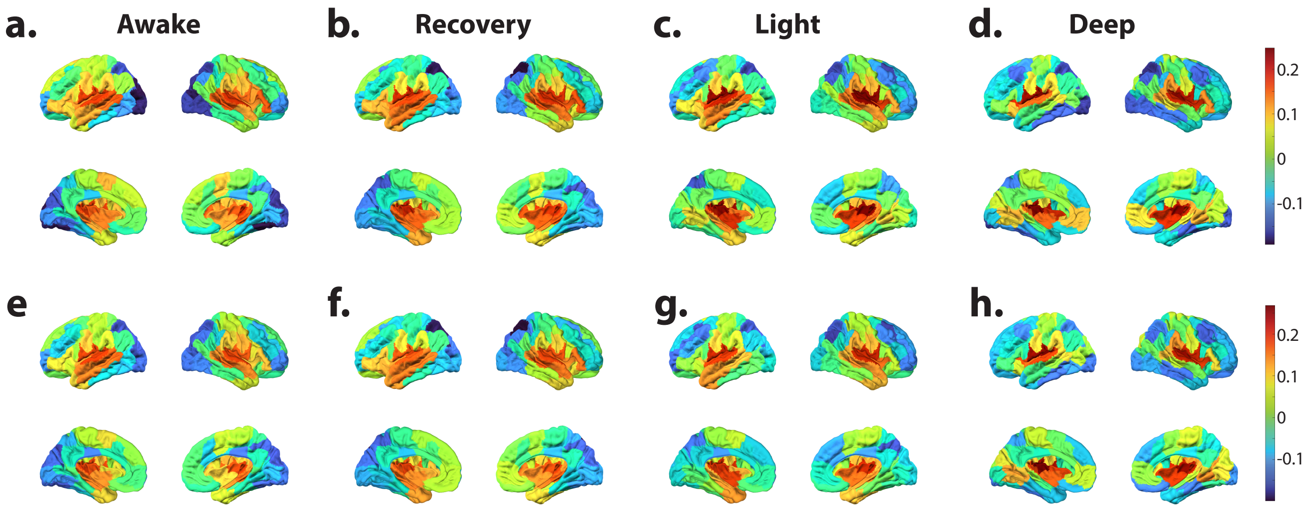

Figure S8. Principal Component Analysis (PCA) of the Inputs' spatial profiles estimated during auditory stimulation paradigm. a-d. The third principal component (PC3) of the Inputs' spatial profiles, estimated using input dimension = 10 and regularization factor = 0.5 during auditory stimulation. PC3 captures the activation of the auditory cortex and other active regions. **e-h.** The third principal component (PC3) of the Inputs' spatial profiles, estimated using input dimension = 25 and regularization factor = 0.5 during auditory stimulation. Note that the spread of coactivation to the higher-order auditory cortices in the temporal lobe during the awake and recovery states is captured using both low and high input dimensions
